# Preparation of a multiepitope vaccine candidate for camel bocavirus and evaluation of its immunogenicity in a mouse model

**DOI:** 10.3389/fimmu.2026.1786028

**Published:** 2026-05-11

**Authors:** Xinyu Tao, Qian Jiang, Yumeng Liang, Xin Li, Xiaojun Ding, Li Yang, Fang Min, Tao Tian, Ruizhen Zhao, Nadiya Duishanbieke, Salamaiti Silajiding, Yi Zhang, Zhanqiang Su, Gang Yao, Xuelian Ma

**Affiliations:** 1 Xinjiang Agricultural University, Urumqi, China; 2 College of Veterinary Medicine, Xinjiang Agricultural University, Urumqi, China

**Keywords:** dromedary camel bocavirus, multi-epitope vaccine, immunoinformatics, humoral immunity, cellular immunity

## Abstract

**Introduction:**

Dromedary camel bocavirus (DBoV) poses a threat to camel health, yet no vaccine is available.

**Methods:**

Using immunoinformatics, B-cell, CTL, and HTL epitopes from VP1/VP2 were predicted. Four multi-epitope constructs (DBoV-A1 to A4) were designed with different adjuvants and evaluated in silico; DBoV-A2 and A4 were expressed, purified, and tested in BALB/c mice.

**Results:**

DBoV-A2 and A4 showed strong in silico binding to TLR9 and stable molecular dynamics. In mice, both induced significant IgG, IgM, IgA responses, Th1/Th2 cytokine secretion, and CD4⁺ T cell expansion without organ toxicity.

**Discussion:**

The consistency between computational predictions and in vivo immunogenicity supports these constructs as promising DBoV vaccine candidates.

## 1 Introduction

As a distinctive livestock sector, the camel industry plays a significant role in promoting economic growth in pastoral areas. In recent years, through policy support and industrial chain extension, some regions of China have promoted the scale-up of camel breeding. However, industrial expansion is accompanied by increased risks of infectious diseases, with high breeding costs, technological lag, and a weak disease prevention and control system emerging as key constraints on development. In this context, dromedary bocavirus (DBoV), as an emerging pathogen, requires increased attention because of its high infection rate and potential for cross-species transmission. This virus belongs to the Parvoviridae family and has a unique ORF3 genomic structure (1). The viral genome is composed of a linear single-stranded DNA approximately 5–5.2 kb in length and encodes the nonstructural proteins NS1 and NP1, as well as the capsid proteins VP1 and VP2. Subsequent studies revealed two genotypes in the Middle East: DBoV1 and DBoV2. Polymerase chain reaction (PCR) analysis revealed that DBoV1 positivity in 93 (13.9%) adult camels and 24 (33.3%) young camels, whereas DBoV2 exhibited positivity in 47 (7.0%) adult camels and 18 (25.0%) young camels (1). Subsequent research data have indicated a high infection rate of DBoV in dromedary camel populations, with particularly prominent rates among younger individuals. Studies suggest that bocaviruses can achieve cross-species transmission. Since human bocavirus (HBoV) was first identified in respiratory samples from children by Swedish researchers in 2005 (2), this virus has been detected in various animals, including gorillas (3), alpacas (4), cats (5), dogs (6), bats (7), pigs (8), rabbits (9), and sea lions (10). Currently, the pathogenic mechanisms of this virus remain unclear, and effective prevention and control measures are still inadequate. Given the high infection rate in camel populations and its potential cross-species transmission risk, the development of safe and effective vaccines has become an urgent research priority.

The advent of reverse vaccinology has significantly advanced the identification of candidate antigen genes and enabled high-throughput genomic screening for immunoprotective antigens. The core of this strategy lies in the application of genetic engineering and recombinant expression technologies to achieve precise design and assembly of selected antigenic epitopes (11–13). In this process, immunoinformatics techniques can effectively identify conserved surface proteins with high species specificity and strong immunogenicity (12). Furthermore, through methods such as gene splicing or linker design, multiple antigenic epitopes derived from different pathogen proteins can be efficiently concatenated to construct multi-epitope fusion antigens, ultimately leading to the development of multi-epitope vaccines (13). Such multi-epitope vaccines can integrate key immunogenic epitopes from different pathogen populations, demonstrating significant potential for the development of broad-spectrum and highly effective vaccines. Their advantages are primarily reflected in two aspects: On the one hand, they can effectively counteract immune evasion caused by antigenic variation, thereby enhancing the comprehensiveness and durability of the immune response (13–15); on the other hand, by covering a greater number of conserved epitopes, this strategy expands the scope of immune protection conferred by the vaccine and improves its ability to defend against different subtypes or variant strains. Consequently, multiepitope vaccines hold profound application prospects in the prevention and control of infections caused by complex pathogens (14–16). Liu et al. developed multiepitope subunit vaccines, MEAS 1 and MEAS 3, which provided broad cross-protection both ex vivo and *in vivo*, effectively reducing the incidence of *Streptococcus suis* infection (17). Yuan et al. developed a broad-spectrum multiepitope vaccine against seasonal influenza A and B viruses in mice that demonstrated significant protective effectiveness against seasonal influenza virus strains in a mouse model, reducing viral load and inflammatory cell infiltration while increasing T-cell and B-cell responses (18). Zeb et al. designed a multiepitope peptide vaccine against Newcastle disease virus (NDV) and demonstrated through molecular dynamics simulations and experimental validation that it can elicit a robust immune response (19). Miao et al. developed a PiuA–PlyD4 fusion protein, which exhibited favourable immunogenicity and immunoreactivity in both immunoinformatic predictions and *in vivo* and ex vivo experiments, demonstrating effectiveness against *Streptococcus pneumoniae* infection (20). Epitope-based vaccine design has initiated a new era in vaccinology and offers an effective strategy for the development of a vaccine against camelid herpesvirus.

As a newly discovered pathogen, Dromedary camel bocavirus (DBoV) poses a potential threat to camel health, yet no effective commercial vaccine is currently available. Given the limited research foundation on this virus and the lengthy development cycle of traditional vaccines, there is an urgent need to explore novel vaccine design strategies. The advancement of reverse vaccinology and immunoinformatics offers a feasible approach for rapid screening and precise vaccine design. Bioinformatics served as the core driver in the multi-epitope vaccine design of this study, with its importance extending throughout the entire process from epitope screening to immunogenicity validation. First, during the epitope screening stage, pan-specific prediction tools such as NetMHCpan-4.1 and NetMHCIIpan-2.1 were employed to systematically identify high-affinity CTL and HTL epitopes targeting BoLA alleles. Concurrently, B-cell linear epitopes and conformational epitopes were predicted using the ABCpred and IEDB ElliPro platforms, enabling the rapid, high-throughput acquisition of immunogenic targets from the viral structural proteins VP1/VP2. Second, in the multi-dimensional safety assessment, candidate epitopes were analyzed for antigenicity, allergenicity, and toxicity via VaxiJen, AllerTOP, and ToxinPred. This ensured that the finally selected epitopes possessed immunostimulatory potential while posing no potential safety risks, thereby completing safety screening at the design stage. Third, in vaccine construction and structural validation, epitopes and adjuvants were rationally assembled using linker peptides (AAY, GPGPG, KK) (15, 21, 22). Subsequently, tertiary structure prediction was performed using I-TASSER, followed by structural optimization with GalaxyRefine and stereochemical quality assessment via Ramachandran plots. The interaction between the vaccine and the TLR9 receptor was then validated through molecular docking (ClusPro) and molecular dynamics simulations, which untangled the immune enhancement mechanism at the atomic level. Finally, the C-ImmSim server was utilized to simulate the dynamics of the immune response, predicting trends in B-cell and T-cell proliferation as well as cytokine secretion, thereby providing a theoretical basis for subsequent animal experiments. This systematic bioinformatics strategy not only significantly enhanced the efficiency and scientific rigor of multi-epitope vaccine development but also established a referential computational framework for vaccine design against other pathogens.

## 2 Materials and methods

**Figure 1:**
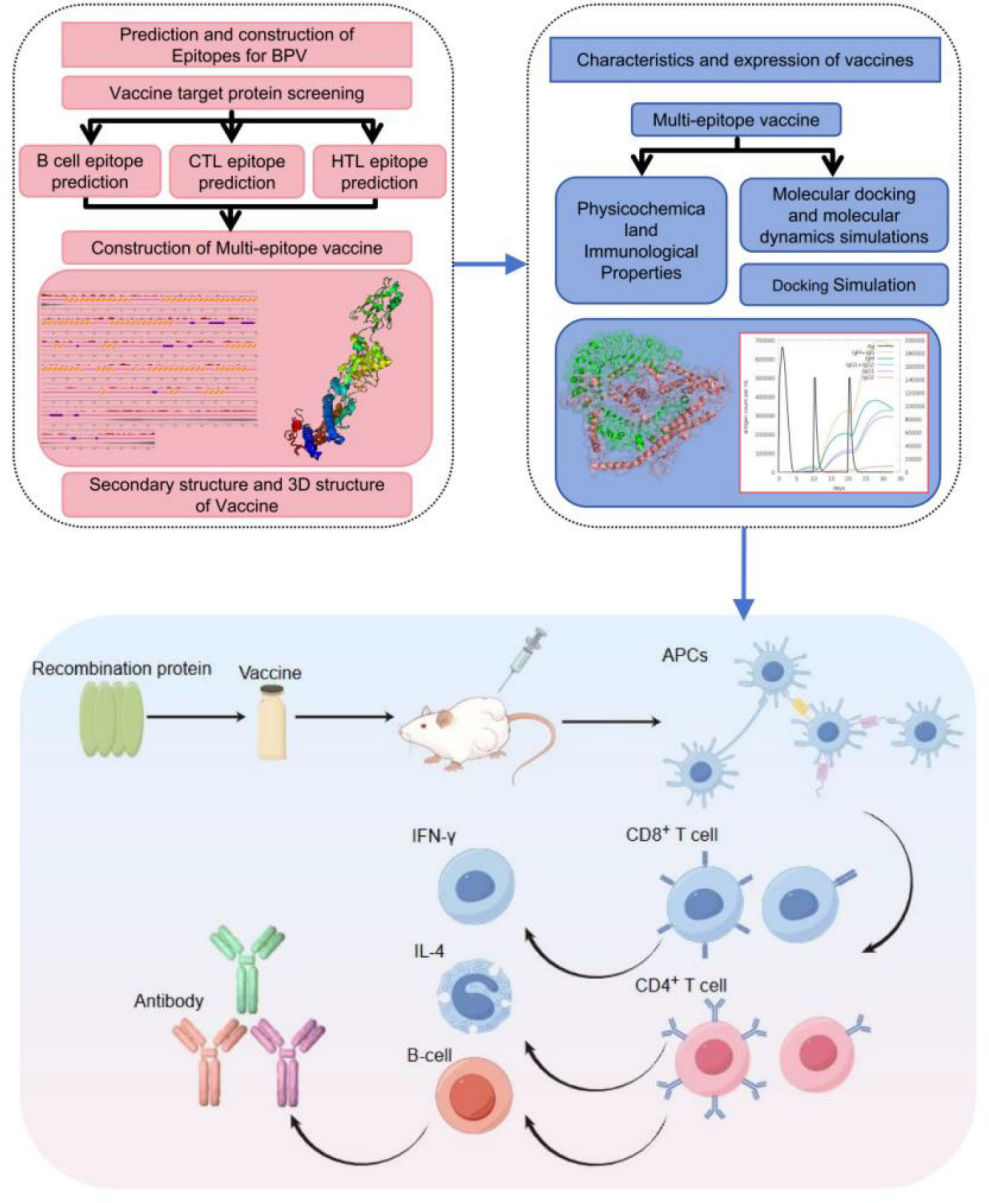
Schematic workflow of multi-epitope vaccine development.The pipeline integrates three stages (1): Computational design – Prediction of B−cell, CTL, and HTL epitopes from DBoV1 VP1/VP2 (ABCpred, NetMHCpan, NetMHCIIpan), assembly with four adjuvants (β-defensin−3, HBHA, L7/L12, FliC) via linkers (EAAAK, AAY, GPGPG, KK) to generate four constructs (DBoV−A1 to A4), and in silico evaluation of antigenicity, allergenicity, and physicochemical properties (2). Structural validation – Tertiary structure prediction (I−TASSER), refinement (GalaxyRefine), validation (Ramachandran, ProSA, ERRAT), mapping of conformational B−cell epitopes (ElliPro), and molecular docking/molecular dynamics with TLR9 (3). Experimental verification – Expression in E. coli, purification, and assessment of safety (histology) and immunogenicity (ELISA, flow cytometry) in BALB/c mice for top candidates DBoV−A2 and A4.

In this study, computer simulations and animal experiments were performed to comprehensively analyse the design of a multiepitope vaccine for DBoV, and the approach are presented in **Figure 1**.

### The retrieval of protein sequences

The VP1 and VP2 protein sequences from 23 distinct DBoV strains (**Supplementary Table 1**) were retrieved in FASTA format from the National Center for Biotechnology Information (NCBI) protein database (https://www.ncbi.nlm.nih.gov/protein). The antigenicity of the reference sequences was evaluated via VaxiJen v.2.0 (https://ddg-pharmfac.net/vaxijen/VaxiJen/VaxiJen.htmI) (23). Strains with an antigenicity score greater than 0.5 were subjected to topology prediction of their protein sequences using TMHMM Server v.2.0 (http://www.cbs.dtu.dk/services/TMHMM/) (24) to identify the most effective antigenic proteins for further analysis.

### Prediction of B-cell epitopes

The antigenic consensus sequences were analysed with the ABCpred server (https://webs.iiitd.edu.in/raghava/abcpred/) to identify antigens that can trigger the production of antibodies by eliciting a B-cell immune response. The server predicts linear B-cell epitopes using an artificial neural network (25). The potential epitopes were screened on the basis of the following prediction parameters: a window length of 16 and a threshold value of 0.5.

### Prediction of cytotoxic T-cell epitopes

In this study, BoLA HLA alleles were considered instead of Camel HLA alleles because of the unavailability of applicable data. Consequently, cattle-related data were utilized to predict the MHC epitopes of selected sequences. In this study, NetMHCpan-4.1 (http://www.cbs.dtu.dk/services/NetCTL/) was employed to predict the CTL epitopes of DBoV candidate antigenic proteins (26). Epitopes with high sensitivity and specificity for BoLA supertypes were selected and entered into IEDB (https://nextgen-tools.iedb.org/pipeline?tool=tc1) for immunogenicity prediction, and CTL epitopes with positive immunogenicity scores were identified (27). The dominant BoLA alleles (BoLA-HD6, BoLA-JSP.1, BoLA-T2c, BoLA-T2b, BoLA-T2a, BoLA-D18.4, BoLA-AW10, and BoLA-T5) were selected for our research because they represented *Bos taurus*, *Bos taurus indicus*, and hybrid bovine species (21).

### Helper T lymphocyte epitope prediction

We used NetMHCII-2.1 (https://services.healthtech.dtu.dk/services/NetMHCIIpan-2.1/) to preliminarily predict the HTL epitopes of DBoV candidate antigen proteins with a threshold of 0.5 (28) and a strong binding site to the BoLA-DRB3 allele (BoLA-DRB3*1501, BoLA-DRB3*0101, BoLA-DRB3*1101, BoLA-DRB3*14011, and BoLA-DRB3*1201) with high sensitivity and specificity (21, 29). The IFN was subsequently compared with the nonIFN predictive model using the IFN epitope tool (http://crdd.osdd.net/raghava/ifnepitope/) in combination with the support vector machine (SVM) method (14). In addition, epitopes induced by IL-4 were identified using the IL4pred tool (http://crdd.osdd.net/raghava/il4pred/) (30).

### Immunoinformatic screening, construction, and characterization of multi-epitope vaccines

Candidate epitopes were evaluated for immunogenic potential and safety prior to vaccine assembly. Antigenicity was assessed using VaxiJen v2.0 (https://ddg-pharmfac.net/vaxijen/VaxiJen/VaxiJen.htmI) with a threshold of >0.5 (23). This threshold corresponds to the default value of the virus model in VaxiJen v2.0, which has been validated to achieve 70–89% prediction accuracy for viral antigens. Allergenicity and toxicity were predicted using AllerTOP v2.0 (https://ddg-pharmfac.net/allertop_test/) (31, 32) and ToxinPred (https://webs.iiitd.edu.in/raghava/toxinpred/design.php) (33), respectively, to select non-allergenic, non-toxic epitopes for further construction. To construct the vaccines, these epitopes were linked to the adjuvant and to the appropriate linking peptides. The CTL epitopes were linked to each other via an AAY linker; likewise, the HTL epitopes and B cell epitopes were linked via GPGPG and KK linkers, respectively. Four adjuvants—beta-defensin-3 (AAV41025.1), HBHA (WP_094028633.1), 50S ribosomal protein L7/L12 (WP_003403353), and Salmonella enterica flagellin FliC (ENT8799295.1)—were each fused via an EAAAK linker to enhance immunogenicity (34). A Pan HLA-DR-binding epitope (PADRE) sequence was also incorporated to improve vaccine stability (15).The final vaccine constructs were re-evaluated for antigenicity using VaxiJen v2.0. Physicochemical properties, including molecular weight, theoretical pI, aliphatic index, grand average of hydropathicity (GRAVY), and instability index, were calculated using ExPASy ProtParam (http://web.expasy.org/protparam/) (35). Allergenicity of the complete constructs was reassessed with AllerTOP v2.0, and solubility was predicted using Protein-Sol (https://protein-sol.manchester.ac.uk) (36).

### Secondary structure prediction and tertiary structure refinement and validation

The NetSurfP-3.0 (https://services.healthtech.dtu.dk/services/NetSurfP-3.0/) server was chosen to predict the secondary structure characteristics of the vaccine (37); the I-TASSER (https://zhanggroup.org/I-TASSER/) service area was used to predict the tertiary structure of the vaccine, and the appropriate model was selected according to its confidence score (C score, typical range: -5, 2) (38). The tertiary structure of the vaccine was optimized by using GalaxyRefine (http://galaxy.seoklab.org/) (39). After refinement, the optimal model was predicted by using ProSA (https://prosa.services.came.sbg.ac.at/prosa.php) and SAVES v6.1 (http://services.mbi.ucla.edu/) (40–42).

### Conformational prediction of the B-cell epitopes

Without adjusting any prediction parameters, conformational epitopes to be recognized by B-cell receptors (BCRs) were predicted using the ElliPro (http://tools.iedb.org/ellipro/) server in the vaccine model (43). B-cell conformational epitopes, which can increase immunogenic reactions, should be present in the vaccine formulation for it to be successful as a preventative regimen.

### Molecular docking and molecular simulation against TLR-9

Cluspro2.0 (http://cluspro.bu.edu/login.php) is an automatic and efficient rigid-body protein docking server can be used to predict protein–protein interactions (44). The best optimized tertiary model structure of the multiepitope vaccine was chosen. Molecular dockings between the vaccine and Bovine Toll-like receptor 9 (TLR9, PDB ID 3WPE) were performed by the Cluspro2.0 server with all parameters set to their default values. The docked structures were visualized by PyMol (45). Molecular dynamics simulation refers to a collection of molecular simulation methods that use Newtonian mechanics to simulate the movement of molecular systems. To investigate the interactions of small molecules with proteins, we performed 100 ns molecular dynamics simulations of the protein–protein complexes using molecular dynamics (46). The root mean square deviation (RMSD), root mean square fluctuation (RMSF), radius of rotation (Rg) and solvent accessible surface area (SASA) data of the molecular dynamics of the protein–protein complexes were obtained.

### Virtual experimentation with the immune system

We simulated and evaluated the immune responses induced by the DBoV-A2 and DBoV-A4 vaccine constructs under a specific immunization protocol using the C-ImmSim server (https://kraken.iac.rm.cnr.it/C-IMMSIM/index.php) (47). All the parameters were assigned their default values except for the removal of lipopolysaccharide (LPS). The vaccination regimen consisted of three immunizations, each containing 1000 units of vaccine protein, with one-month intervals between doses. The simulation predicted the ability of DBoV-A2 and DBoV-A4 to elicit specific antibodies and multiple cytokines and assessed the immune responses of B-cell and T-cell populations.

### Construction, expression, purification and identification of recombinant plasmids

The recombinant plasmids pET30a-DBoV-A2 and pET30a-DBoV-A4, incorporating NdeI and HindIII restriction sites, were synthesized and sequence-verified by GenScript (Nanjing, China). The verified recombinant plasmids were transformed into *E. coli* BL21(DE3), and protein expression was induced with 500 μM IPTG at 16 °C for 16 h. Cells were harvested, resuspended in lysis buffer (20 mM Tris-HCl, 500 mM NaCl, 10 mM imidazole, 1 mM PMSF, pH 8.0), and disrupted by sonication. After centrifugation, the supernatant was loaded onto a Ni-NTA affinity column (Beyotime, China). The column was washed with wash buffer (20 mM Tris-HCl, 500 mM NaCl, 20 mM imidazole, pH 8.0), and the target protein was eluted with elution buffer (20 mM Tris-HCl, 500 mM NaCl, 300 mM imidazole, pH 8.0). The eluted protein was stepwise renatured by dialysis against 6–2 M urea and finally PBS, then concentrated by ultrafiltration. Protein purity was assessed by SDS-PAGE, identity confirmed by Western blot, and concentration determined using a BCA assay (Solarbio, China).

### Animal immunization and sample collection

Thirty female BALB/c mice (6–8 weeks old) were randomly divided into three groups (n = 10 per group): a control group receiving PBS emulsified with Freund’s adjuvant, a DBoV−A2 group immunized with DBoV−A2 fusion protein, and a DBoV−A4 group immunized with DBoV−A4 fusion protein. Each mouse in the experimental groups was injected subcutaneously with 50 μg of the corresponding fusion protein emulsified in Freund’s adjuvant. The immunization schedule comprised a primary dose on day 0, followed by two booster doses on days 14 and 28. Complete Freund’s adjuvant (F5881, Merck, Germany) was used for the primary immunization, and incomplete Freund’s adjuvant (F5506, Merck, Germany) for the booster immunizations. Blood samples were collected from the tail vein on days 0, 14, 28, and 42. Serum was separated by incubation at 37 °C for 2 h and stored at –20 °C until analysis.

On day 42 (14 days after the final immunization), all mice were euthanized by cervical dislocation performed by trained personnel, and death was confirmed by thoracotomy or dissection of vital organs. Organs including the heart, liver, spleen, lungs, and kidneys were collected immediately, fixed in 4% paraformaldehyde (G1101−500ML, Saibo Biology, China), and subsequently embedded in paraffin. Tissue sections (5 μm) were prepared by Saibo Biology (Wuhan, China) for histological examination to evaluate the safety of the vaccine candidates.

### Assessment of immunogenicity

Immunogenicity was evaluated using an indirect ELISA (Enzyme-Linked Immunosorbent Assay). Microplates were pre-coated with DBoV-A2 or DBoV-A4 fusion protein and blocked with PBST (PBS containing 0.05% Tween-20) supplemented with 5% skimmed milk at 37 °C for 2 hours. Subsequently, test serum samples were added and incubated at 37 °C for 1 hour. Horseradish peroxidase (HRP)-conjugated goat anti-mouse IgG (SA00001-1; Proteintech, China), IgA (RGAM801; Proteintech, China), and IgM (RGAM701; Proteintech, China) were used as secondary antibodies (all at a working dilution of 1:5000) and incubated at 37 °C for 1 hour. Color development was performed using TMB substrate at room temperature for 15 minutes in the dark. The reaction was terminated by adding 2 M H_2_SO_4_, and the absorbance was measured at 450 nm.

### Analysis of IL-4 and IFN-γ secretion

Serum IL-4 and IFN-γ levels were quantified using commercial ELISA kits (JONLNBIO, China) following the manufacturer’s protocols. Briefly, 96-well plates pre-coated with capture antibodies were incubated with serum samples (diluted 1:2) or cytokine standards for 2 h at 37 °C. After washing, biotin-conjugated detection antibody was added, followed by HRP-streptavidin. Color was developed with TMB substrate, stopped with 2 M H_2_SO_4_, and absorbance measured at 450 nm. Cytokine concentrations were calculated from standard curves and expressed in pg/mL. All samples were assayed in duplicate at baseline (day 0) and on days 14, 28, and 42 post-immunization.

### Flow cytometry

After the third immunization, the spleen lymphocytes were collected, and the cell density was adjusted to 1 × 10^6^/tube. Fluorescent-labelled antibodies against PE-CD3 (E-AB-F1013D; Elabscience, China), APC-CD4 (E-AB-F1097E; Elabscience, China), and FITC-CD8a (E-AB-F1104C; Elabscience, China) were added, and the cells were incubated at room temperature in the dark for 30 min. Then, the cells were washed with PBS three times and resuspended in 500 μL of PBS. CD3^+^CD4^+^ and CD3^+^CD8^+^ T cells were analysed by a flow cytometer (Mindray, China). Data analysis was performed using FlowJo (v10.8.1).

### Statistical analysis

Data were analyzed using GraphPad Prism software (version 9.0). All datasets were first assessed for normality using the Shapiro-Wilk test and for homogeneity of variance using Levene’s test. The results confirmed that the data from all groups followed a normal distribution (*P* > 0.05) and exhibited homogeneity of variance (*P* > 0.05), thereby satisfying the prerequisites for parametric tests. Differences between groups were evaluated using two-way analysis of variance (two-way ANOVA). Data are presented as mean ± standard deviation. Significance levels are denoted as follows: ns (*P* ≥ 0.05), * (*P* < 0.05), ** (*P* < 0.01), and *** (*P* < 0.001).

## 3 Results

### Selection of target proteins

The sequences of the strains with the highest antigenicity were selected for vaccine development. The antigenicity scores for VP1 (ASC49312.1) and VP2 (ASC49313.1) were 0.5814 and 0.5908, respectively, confirming their suitability as immunogenic targets.

### Prediction of the BCL epitopes

In this study, the ABCpred server was used to predict B-cell linear (BCL) epitopes, and overlapping BCL epitopes with strong antigenicity, no allergenicity, no toxicity, and no mutation tendency were screened. The results revealed that 40 VP1 and 32 VP2 epitopes were identified as antigenic (threshold > 0.5) according to the VaxiJen server. After further analysis for allergenicity, six BCL epitopes were selected, including 3 from the VP1 protein and 3 from the VP2 protein (**Table 1**; **Supplementary Table 2**). These epitopes were incorporated into the vaccine design.

**Table 1:** Final selected linear B-cell epitopes.

Protein	Peptide	Antigenicity	Allergenicity	Toxicity	Mutagenicity
VP1	YLGPFNPLDNGEPVNK	0.9002	Nonallergen	Nontoxin	No Mutation
	VKRALAPSLNEKQLAP	1.0395	Nonallergen	Nontoxin	No Mutation
	YFARSNKGAKRQRLNP	0.9488	Nonallergen	Nontoxin	No Mutation
VP2	ANAGTGGVGMSTGQWI	0.7694	Nonallergen	Nontoxin	No Mutation
	DSINITRYNPIWVKTP	0.6464	Nonallergen	Nontoxin	No Mutation
	EIEWEYETHFNKNWRP	1.276	Nonallergen	Nontoxin	No Mutation

### Prediction of the CTL epitopes

The CTL epitopes (8–11 amino acids) of the two candidate proteins were predicted using the NetCTL 1.2 web server. Epitopes with strong antigenicity, immunogenicity, nonallergenicity, nontoxicity, and nonmutagenicity were selected. The results revealed that 34 VP1 and 12 VP2 epitopes exhibited antigenicity scores greater than 0.5. Following the immunogenicity assessment, these epitopes were narrowed to 14 immunogenic VP1 and 12 immunogenic VP2 epitopes. Ultimately, allergenicity analysis revealed a total of 7 CTL antigenic epitopes, including 4 from the VP1 protein and 3 from the VP2 protein (**Table 2**; **Supplementary Table 3**).

**Table 2:** The scores and antigenicity of the predicted CTL epitopes.

Protein	Peptide	Antigenicity	Immunogenicity	Allergenicity	Toxicity	Mutagenicity	Allele
VP1	YNYLGPFNPL	0.6946	0.1081	Nonallergen	Nontoxin	No Mutation	BoLA-JSP.1
	KQLAPGRSA	0.8431	0.00389	Nonallergen	Nontoxin	No Mutation	BoLA-D18.4 BoLA-T5
	FSPKEWQTL	1.2179	0.01033	Nonallergen	Nontoxin	No Mutation	BoLA-JSP.1 BoLA-AW10
	KVYNIQIK	1.2557	0.12517	Nonallergen	Nontoxin	No Mutation	BoLA-T2a
VP2	FQNDLTAGL	0.616	0.09878	Nonallergen	Nontoxin	No Mutation	BoLA-JSP.1 BoLA-T2c BoLA-T7 BoLA-D18.4
	KQYAYITCPY	0.7575	0.14873	Nonallergen	Nontoxin	No Mutation	BoLA-T5
	MVDTRDGTL	1.5191	0.16882	Nonallergen	Nontoxin	No Mutation	BoLA-T7

### Prediction of the HTL epitopes

HTL epitopes of the two candidate proteins were predicted using the NetMHCII web server. HTL epitopes exhibiting strong antigenicity, immunogenicity, nonallergenicity, nontoxicity, and the absence of mutations were selected. These HTL epitopes were further evaluated for their ability to induce IFN-γ and interleukin responses using IFN epitope and IL4pred immunoinformatic tools. The results demonstrated that a total of 67 VP1 and 39 VP2 T-cell epitopes possessed IFN-γ-inducing properties, whereas only 24 VP1 epitopes and 44 VP2 epitopes exhibited IL-4-inducing characteristics. Finally, antigenicity and allergenicity analyses were performed using the VaxiJen and AllerTop v2.0 servers to screen out nonantigenic and nonallergenic epitopes. Ultimately, three VP1 epitopes and one VP2 epitope were identified as the most promising HTL epitope candidates for the vaccine construct (**Table 3**; **Supplementary Table 4**).

**Table 3:** The percentile ranks and antigenicity of the predicted HTL epitopes.

Protein	Peptide	Antigenicity	Allergenicity	Toxicity	Mutagenicity	Allele
VP1	AYNQYLNKGLNPYLK	0.469	Nonallergen	Nontoxin	No Mutation	BoLA-DRB3_1101
	KLYFARSNKGAKRQR	0.4097	Nonallergen	Nontoxin	No Mutation	BoLA-DRB3_1101 BoLA-DRB3*0101 BoLA-DRB3*14011
	EWQTLLNIAKRFRPV	0.7848	Nonallergen	Nontoxin	No Mutation	BoLA-DRB3_1101 BoLA-DRB3*0101 BoLA-DRB3*14011
VP2	AWDSINITRYNPIWV	1.1852	Nonallergen	Nontoxin	No Mutation	BoLA-DRB3*1501 BoLA-DRB3*14011 BoLA-DRB3_1101

### Construction of four multi-epitope vaccines (DBoV-A1 to A4)

**Figure 2:**
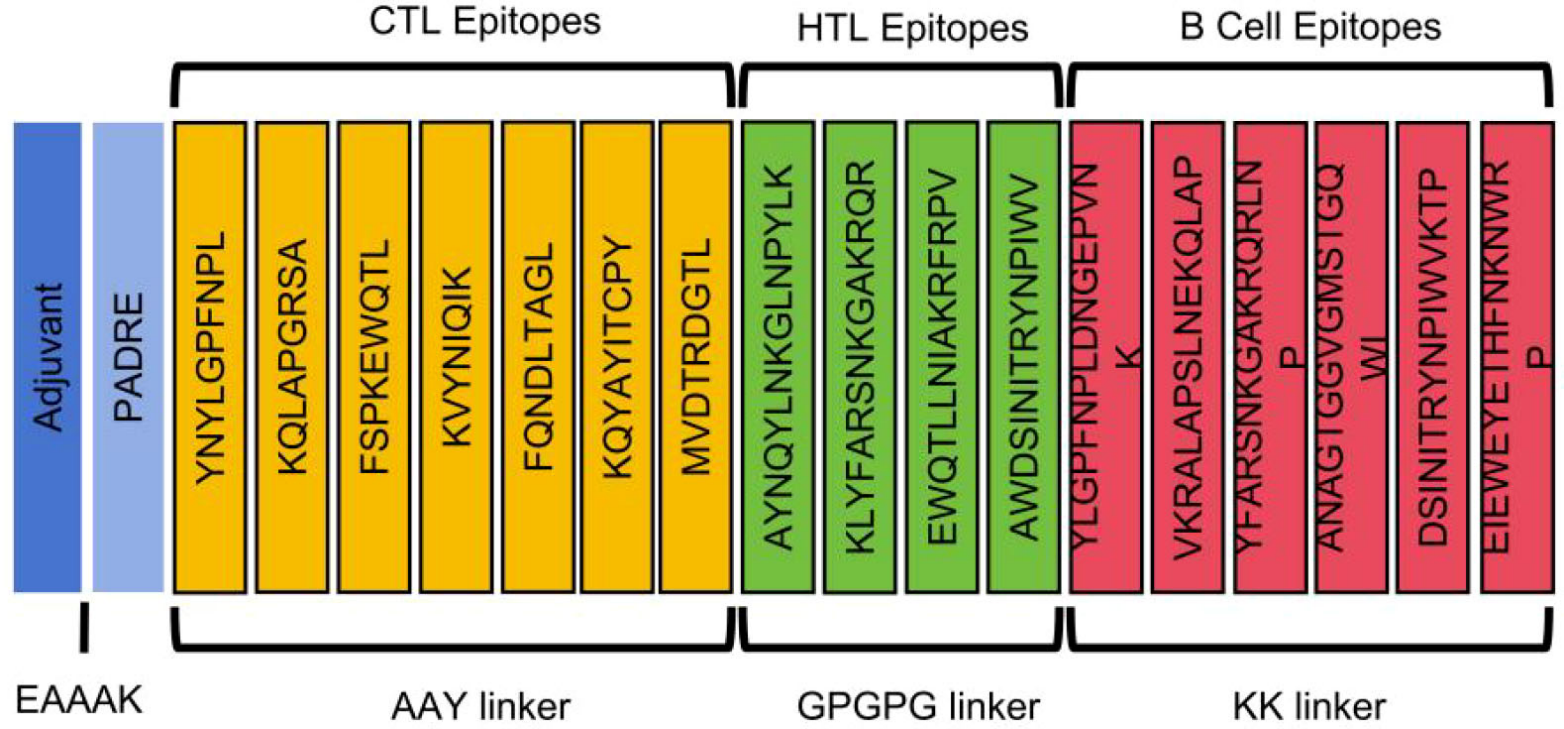
Schematic structure of the multi-epitope vaccine constructs. The four constructs (DBoV−A1 to A4) each contain an adjuvant (β-defensin−3, HBHA, L7/L12, or FliC), the PADRE sequence, and selected B−cell, CTL, and HTL epitopes arranged from N− to C−terminus. Linkers: EAAAK connects the adjuvant to PADRE; AAY links CTL epitopes; GPGPG links HTL epitopes; KK links B−cell epitopes.

A total of 6 BCL epitopes, 7 CTL epitopes, and 4 HTL epitopes were employed to construct the multiepitope vaccines. DBoV-A1, DBoV-A2, DBoV-A3, and DBoV-A4 vaccines were designed, and each included a protein adjuvant (beta-defensin-3, HABA protein, 50S ribosomal protein L7/L12, and *Salmonella enterica* flagellin FliC), a PADRE sequence, and T-cell and B-cell epitopes, as well as the linkers that linked them together (**Figure 2**; **Supplementary Table 5**). The anticipated epitopes were separated by linkers (AAY, GPGPG, and KK). The EAAAK sequence was chosen to connect the initial adjuvant sequences to the PADRE sequence, thereby resulting in the production of the vaccine. The PADRE sequence was used to increase the stability and effectiveness of the peptide vaccines. The vaccine constructs DBoV-A1, DBoV-A2, DBoV-A3, and DBoV-A4 had 336, 450, 421, and 522 residues, respectively.

### Predicted antigenicity, stability, and safety of all constructs

Physicochemical characterization revealed that the instability index of all the vaccine structures was less than 40 and that each vaccine was expected to have a half-life of greater than 10 hours in *E. coli* (*in vivo*) and a negative GRAVY value. The four multiepitope vaccines had an antigen index greater than 0.5 and a solubility greater than 0.5. The four multiepitope vaccines described above included nonallergic, highly soluble antigenic proteins (**Table 4**).

**Table 4:** Evaluation of the allergenicity, antigenicity, solubility, and physicochemical properties of the vaccine constructs.

Features	DBoV-A1	DBoV-A2	DBoV-A3	DBoV-A4
Amino acid number (aa)	336	450	421	522
Molecular weight	37650.53	50117.98	45929.82	57156.44
Theoretical isoelectric point (pI)	10.1	9.68	9.64	9.73
Aliphatic index	65.51	74.38	77.15	75.08
Grand average of hydropathicity (GRAVY)	-0.677	-0.611	-0.41	-0.598
Instability index	38.71	39.04	31.37	28.59
Antigenicity	0.7057	0.6414	0.6187	0.5841
Solubility	0.635	0.581	0.591	0.551
Allergenicity	Nonallergen	Nonallergen	Nonallergen	Nonallergen

### DBoV-A2 and DBoV-A4 show superior structural quality

**Figure 3:**
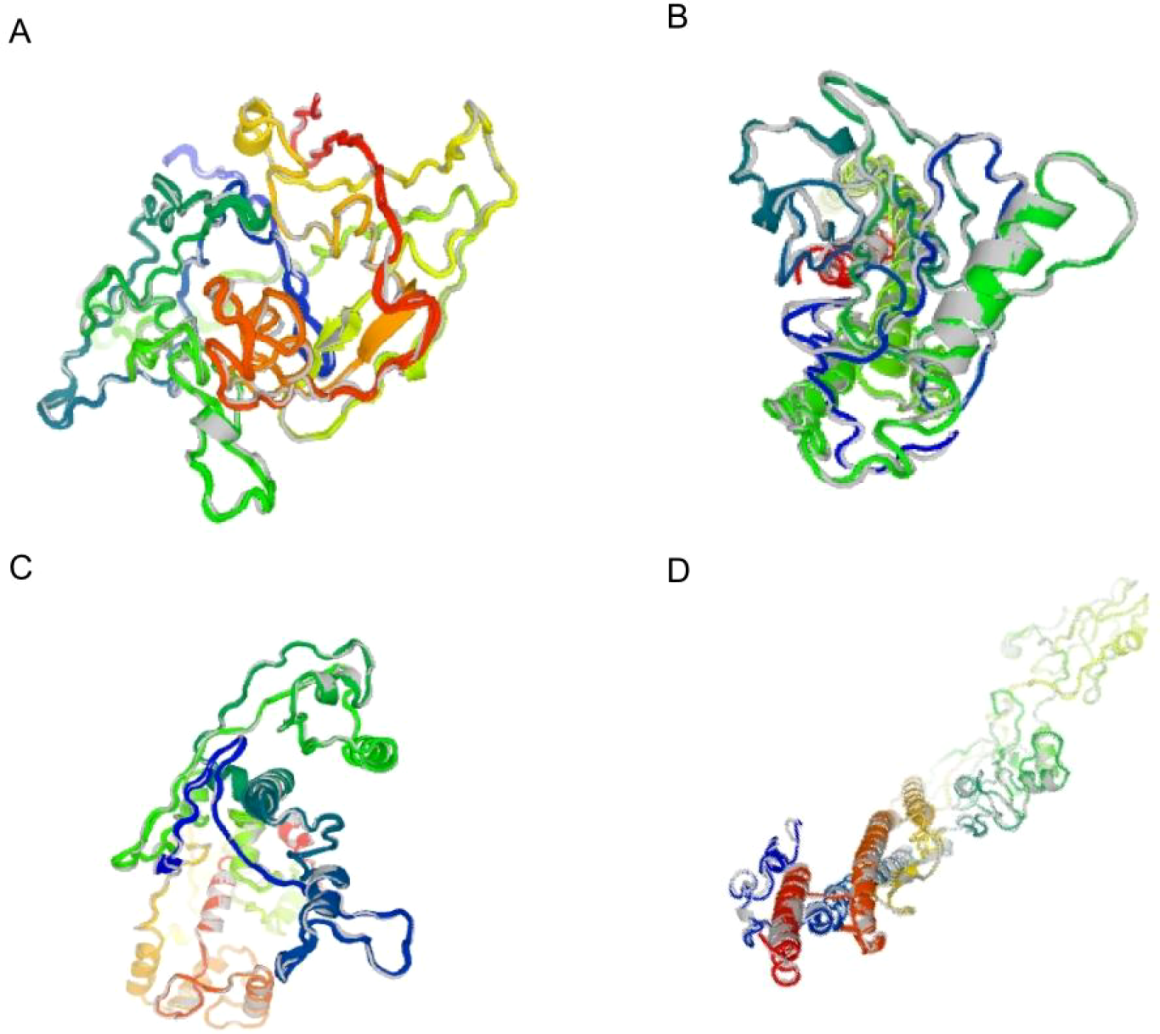
Tertiary structure plot of the epitope vaccines. **(A)** DBoV-A1; **(B)** DBoV-A2; **(C)** DBoV-A3; **(D)** DBoV-A4.The structural quality was validated by Ramachandran plot analysis (see **Supplementary Figure 2** for detailed plots; key parameters are summarized in **Table 5**).

NetSurfP-3.0 analysis revealed that DBoV-A1 to A4 contained 25.6–51.3% alpha helices, 0–6.13% beta turns, and 48–69.1% random coils, indicating stable secondary structures (**Supplementary Figure 1**, **Supplementary Table 6**). Furthermore, the tertiary structures of the vaccines were generated by the I-TASSER server on the basis of C-scores (**Table 5**, **Figure 3A–D**; **Supplementary Figure S2**). The C-score typically ranges from [–5, 2], with higher values indicating greater model confidence. The results showed that all four models had C-scores within the [–5, 2] range, indicating high model reliability. The validity of the three-dimensional models was verified using the SAVE server based on Ramachandran rules. The results indicated that 60%–90% of the amino acid residues across all four vaccines were located in favored regions. Specifically, Ramachandran plot analysis revealed that 87.6% of residues in DBoV-A2 and 80.3% of residues in DBoV-A4 were situated in the most favored regions (**Table 5**; **Supplementary Figure 2**), suggesting good stereochemical quality. The overall and local quality of the three-dimensional vaccine models was assessed by the ProSA-web service using Z scores (**Table 5**). The results indicated that both the Ramachandran plot statistics and Z scores of DBoV-A2 and DBoV-A4 were greater than those of the other vaccines. Consequently, DBoV-A2 and DBoV-A4 were selected for subsequent investigation.

**Table 5:** Structural quality comparison of the modelled vaccine constructs.

Properties	DBoV-A1	DBoV-A2	DBoV-A3	DBoV-A4
C-score	-2.31	-1.86	-1.48	-1.93
ERRAT	48.0769	80.3874	79.4286	79.2899
Ramachandran plot	68.10%	87.60%	79.80%	80.30%
Distribution Z score	-3.45	-5.02	-3.19	-6.03

### Conformational B-cell epitopes confirmed in DBoV-A2/A4

Ellipro software predicted B-cell conformational epitopes for both DBoV-A2 and DBoV-A4. DBoV-A2 contained 213 residues, with scores between 0.514 and 0.806, while DBoV-A4 contained 258 residues with scores ranging from 0.529 to 0.827. The data in **Supplementary Figure 2**; **Table 6** reveal that the DBoV-A2 epitopes spanned 3 to 75 amino acid residues, whereas **Supplementary Figure 3**; **Table 7** present the DBoV-A4 epitopes ranging from 3 to 96 residues. The combination of extensive residue counts and elevated scores strongly supports the immunogenic potential of the multiepitope vaccines.

**Table 6:** Conformational B-cell epitopes of DBoV-A2.

Serial number	Residues	Number of residues	Score
1	K74, F75, T76, T77, E78, E79, L80, R81, K82, A83, A84, E85, G86, Y87, L88, E89, A90, A91, T92, N93, Y95, N96, E97, L98, V99, E100, R101, G102, E103, A104, A105, L106, Q107, R108, L109, R110, S111, Q112, T113, A114, F115, E116, D117, A118, S119, A120, R121, A122, E123, G124, Y125, V126, D127, Q128, A129, V130, E131, L132, T133, Q134, E135, A136, L137, G138, T139, V140, A141, S142, Q143, T144, R145, A146, V147, G148, E149	75	0.806
2	K379, K380, Y381, F382	4	0.795
3	F291, A292, R293, S294, N295, K296, G297, A298, K299, R300, Q301, R302, G303, P304, G305, P306, G307, E308, T311, N314, I315, A316, K317, R318, F319, R320, P321, V322, G323, P324, G325, P326, G327, A328, W329, K415, K416, D417, S418, I419, N420, I421, T422, R423	44	0.747
4	N278, P279, Y280, L281, K282	5	0.733
5	I246, T247, C248, P249, Y250, A251, A252, Y253, D256, T257, R258, N445, K446, W448	14	0.693
6	Y245, G283, P284, G285, P286, G287, K288, L289, Y290, T335, R336, W438, Y440, E441, T442, H443, F444	17	0.672
7	W341, V342, K344, Y345, L346, G347, P348, F349, N350, P351, L352, D353, N354, G355, E356, A368, P369, S370, L371, N372, E373, K374, Q375, L376, A377, P378, A383, R384, N386, Y424, N425	31	0.651
8	F68, I69, L71, R72, D73	5	0.644
9	G199, A202, A203, F206	4	0.607
10	A389, K390, R391	3	0.599
11	D330, S331, N333, V406, G407, M408, S409, T410, G411, Q412, I414	11	0.514

**Table 7:** Conformational B-cell epitopes of DBoV-A4.

Serial number	Residues	Number of residues	Score
1	M1, A2, Q3, V4, I5, N6, T7, N8, S9, L10, S11, L12, L13, T14, Q15, N16, N17, L18, N19, K20, S21, Q22, S23, S24, L25, S26, S27, A28, I29, E30, R31, L32, S33, S34, G35, L36, R37, I38, N39, S40, A41, K42, D43, D44, A45, A46, G47, Q48, A49, I50, N52, G483, Q484, W485, K487, K488, D489, S490, I491, N492, I493, T494, R495, Y496, N497, P498, I499, W500, V501, K502, T503, P504	72	0.827
2	Y199, A200, A201, G202, A203, D204, K205, Y206, R207, V208, D209, I210, N211, S212, G213, A214, V215, V216, T217, D218, A219, A220, A221, P222, D223, K224, V225, Y226, V227, N228, A229, A230, N231, E232, A233, A234, A235, K236, A237, K238, F239, V240, A241, A242, W243, T244, L245, K246, A247, A248, A249, A250, A251, Y252, Y253, N254, Y255, L256, G257, P258, F259, N260, P261, L262, A263, A264, Y265, K266, L268, A269, P270, G271, R272, S273, A274, A275, A276, Y277, F278, S279, P280, K281, E282, W283, Q284, T285, L286, A287, A288, Y289, K290, V291, Y292, N293, I294, Q295	96	0.759
3	K505, K506, E507, I508, E509, W510, E511, Y512, E513, T514, H515, F516, N517, K518, N519, W520, R521, P522	18	0.703
4	V92, R93, E94, L95, S96, V97, Q98, A99, T100, N101, G102, T103, N104, S105, D106, S107, D108, L109, S111, I112, G169, G172, N174, V175, N176, G177, P178, K179, E180, A181, T182, V183, G184, P321, Y322, A323, A324, Y325, M326, V327, D328, T329, R330, D331, G332, T333, G397, P398, K434, V435, K436, A438, L439, A440, P441, S442, L443, N444, E445, K446, Q447	61	0.64
5	K432, K433, R437	3	0.633
6	A449, P450, Y453	3	0.582
7	K313, Q314, Y315, Y341, N342	5	0.529

### DBoV-A2 and DBoV-A4 bind stably to TLR9

**Figure 4:**
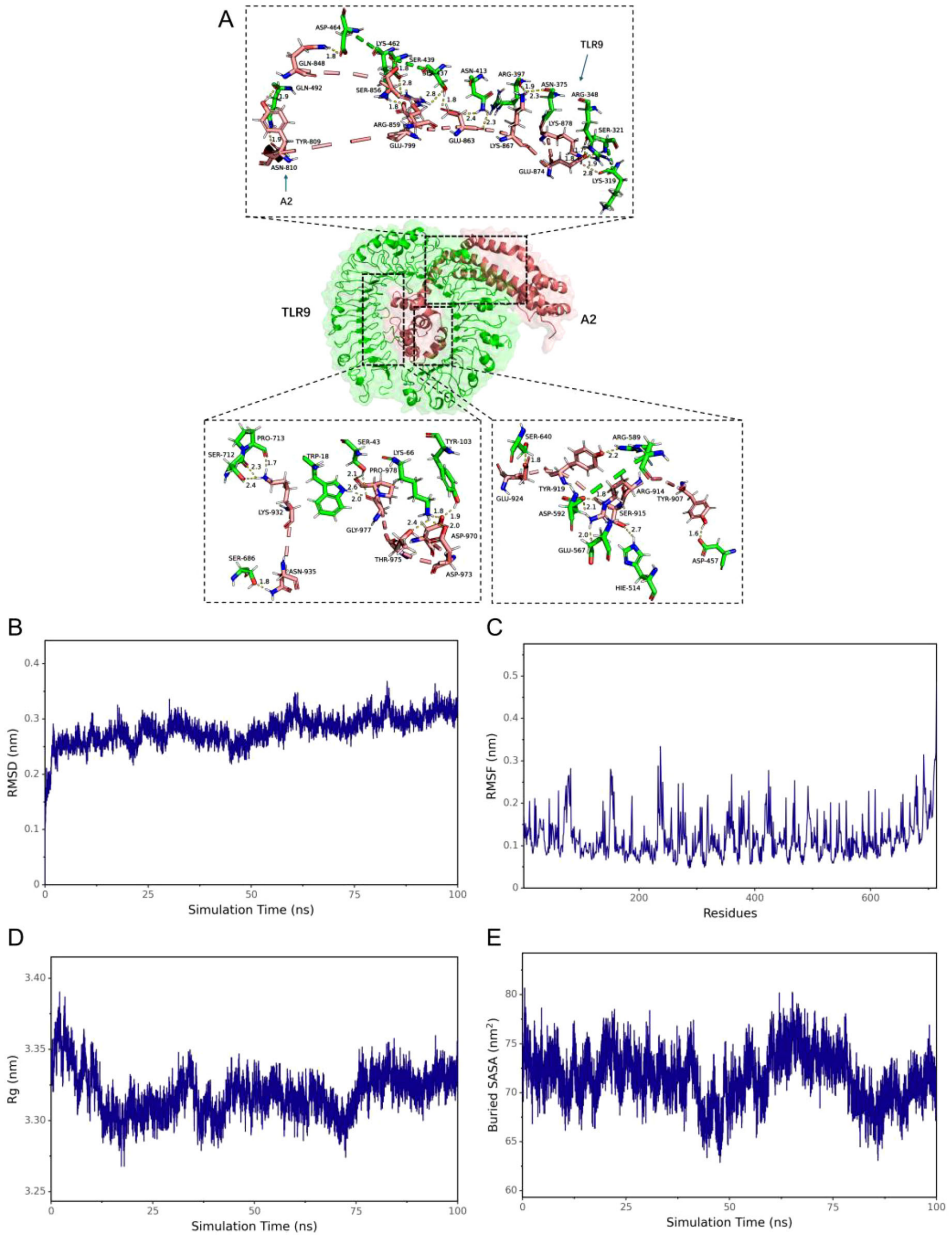
Molecular docking results of the DBoV-A2–TLR9 complex. **(A)** Outcome of the molecular docking of DBoV-A2 with TLR9. **(B–E)** Molecular dynamics simulations of the DBoV-A2–TLR9 complex, including RMSD values of the complex backbone, RMSF values of the side chain residues, radius of gyration, and solvent accessible surface area (SASA) during the molecular dynamics simulations.

**Figure 5:**
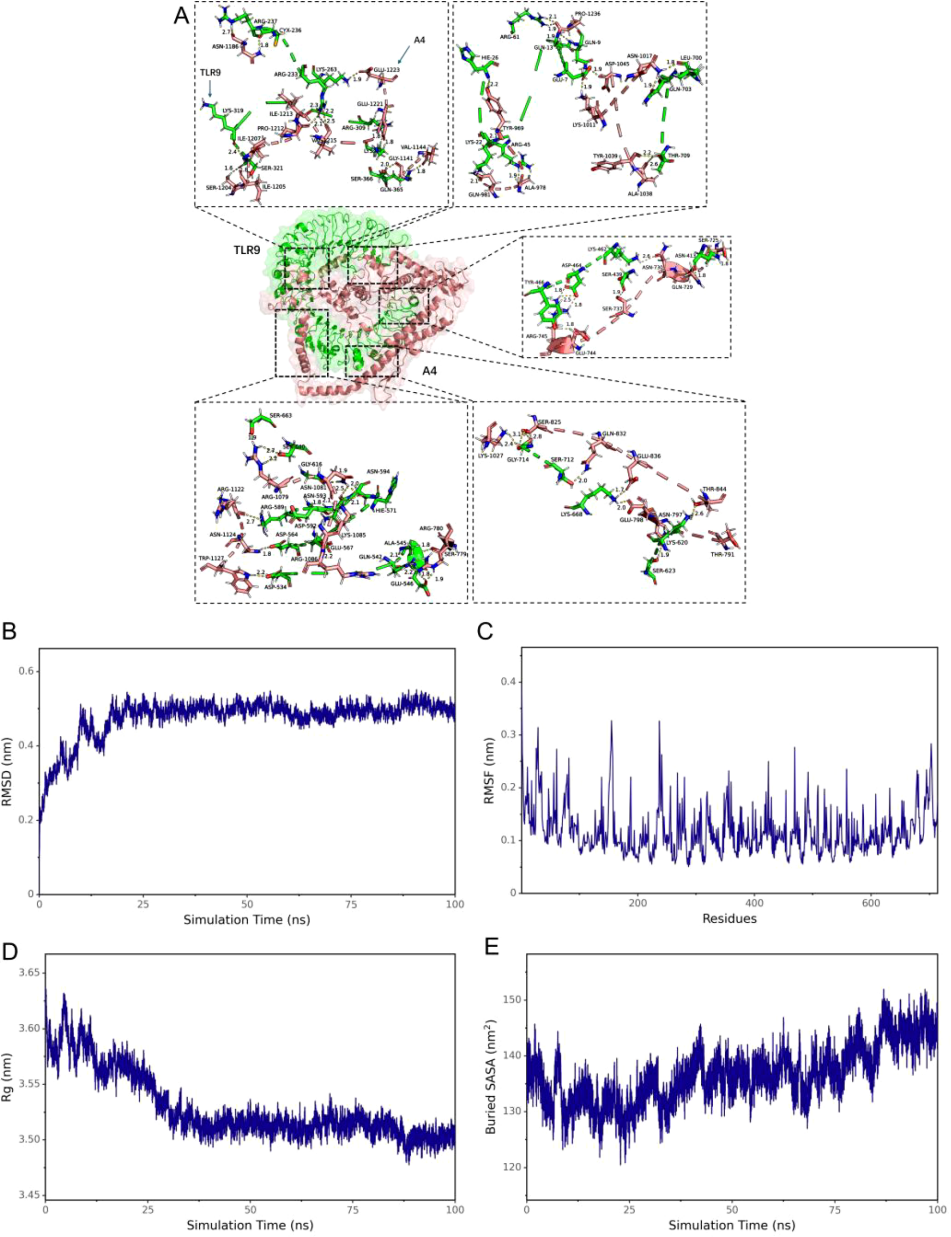
Molecular docking results of the DBoV-A4–TLR9 complex. **(A)** Outcome of the molecular docking of DBoV-A4 with TLR9. **(B–E)** Molecular dynamics simulations of the DBoV-A4–TLR9 complex, encompassing RMSD values of the complex backbone, RMSF values of the side chain residues, radius of gyration, and solvent accessible surface area (SASA) during the simulations.

For molecular docking simulations, we docked the fine tertiary structure of the multiepitope vaccine with toll-like receptor 9 (TLR 9) using ClusPro 2.0. Each receptor–ligand complex produced 30 different docking poses, which exhibited different orientations. Given that lower energy scores indicate greater binding affinity, the most favourable docked complex was identified by the lowest energy score. The energy scores for the DBoV-A2–TLR9 complex and DBoV-A4–TLR9 complex were -357.587 ± 12.209 kJ/mol and -748.887 ± 46.734 kJ/mol, respectively. The topological structure and binding interactions of the DBoV-A2–TLR9 complex are shown in **Figure 4A**, whereas those of the DBoV-A4–TLR9 complex are shown in **Figure 5A**.

Molecular dynamics simulations of the docked vaccine–TLR9 complexes were performed with the GROMACS software package. These simulations were executed for a duration of 100 nanoseconds (ns) to estimate the dynamic behaviour and stability of the complexes. Subsequent analysis of the generated trajectory data yielded calculations of the root mean square deviation (RMSD) of the protein backbone, root mean square fluctuation (RMSF) of the side chains, radius of gyration (Rg), and solvent accessible surface area (SASA). The RMSD, RMSF, Rg, and SASA values for the DBoV-A2–TLR9 and DBoV-A4–TLR9 complexes are shown in **Figures 4B–E**, **5B–E**, respectively, which confirm the relative stability of the docked complexes.

### Immune simulation predicts robust responses to DBoV-A2/A4

**Figure 6:**
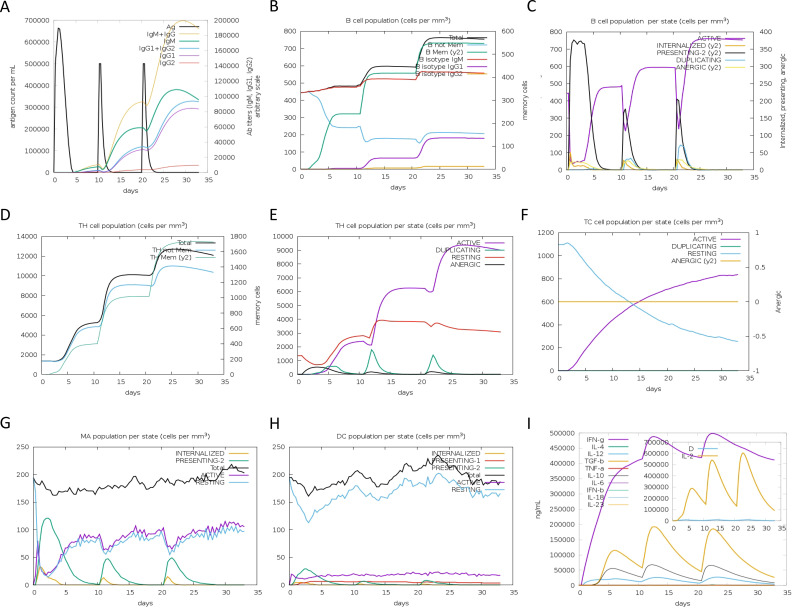
In silico simulation of the immune response with DBoV-A2 as the antigen. **(A)** Antigen and immunoglobulins, with antibodies subdivided by isotype. **(B–F)** B-cell populations, B-cell counts, helper T-cell populations, helper T-cell counts per state, and cytotoxic T-cell counts per state. **(G–H)** Macrophage and dendritic cell populations per state. **(I)** Cytokine with D indicating a danger signal.

**Figure 7:**
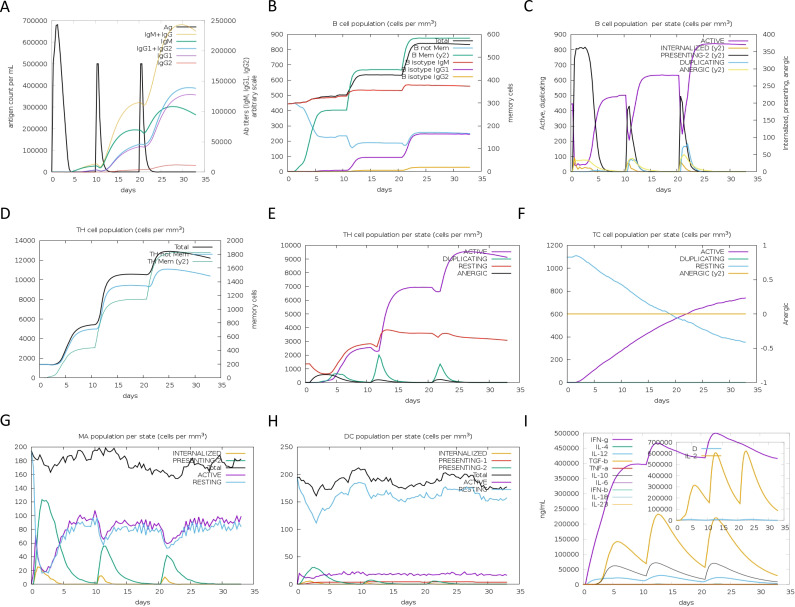
In silico simulation of the immune response with DBoV-A4 as the antigen, following the same layout as that of **Figure 6**.

To evaluate the immunogenic spectrum of the multiepitope vaccines, we conducted in silico immunization simulations using the C-ImmSim server. The results indicated prominent immunoglobulin activity for both DBoV-A2 and DBoV-A4 in terms of the immune response (**Figures 6A**, **7A**). High levels of B-cell, helper T-cell, and cytotoxic T-cell activity were observed in the immunization process (**Figures 6B–F**, **7B–F**). Macrophage and dendritic cell activity also rapidly increased after each exposure (**Figures 6G, H**, **7G, H**). Additionally, elevated levels of INF-γ, IL-2, and IL-12 were detected (**Figures 6I**, **7I**). This immunogenic profile suggests that the multiepitope vaccines can induce effective immune responses.

### Successful expression and purification of DBoV-A2/A4

**Figure 8:**
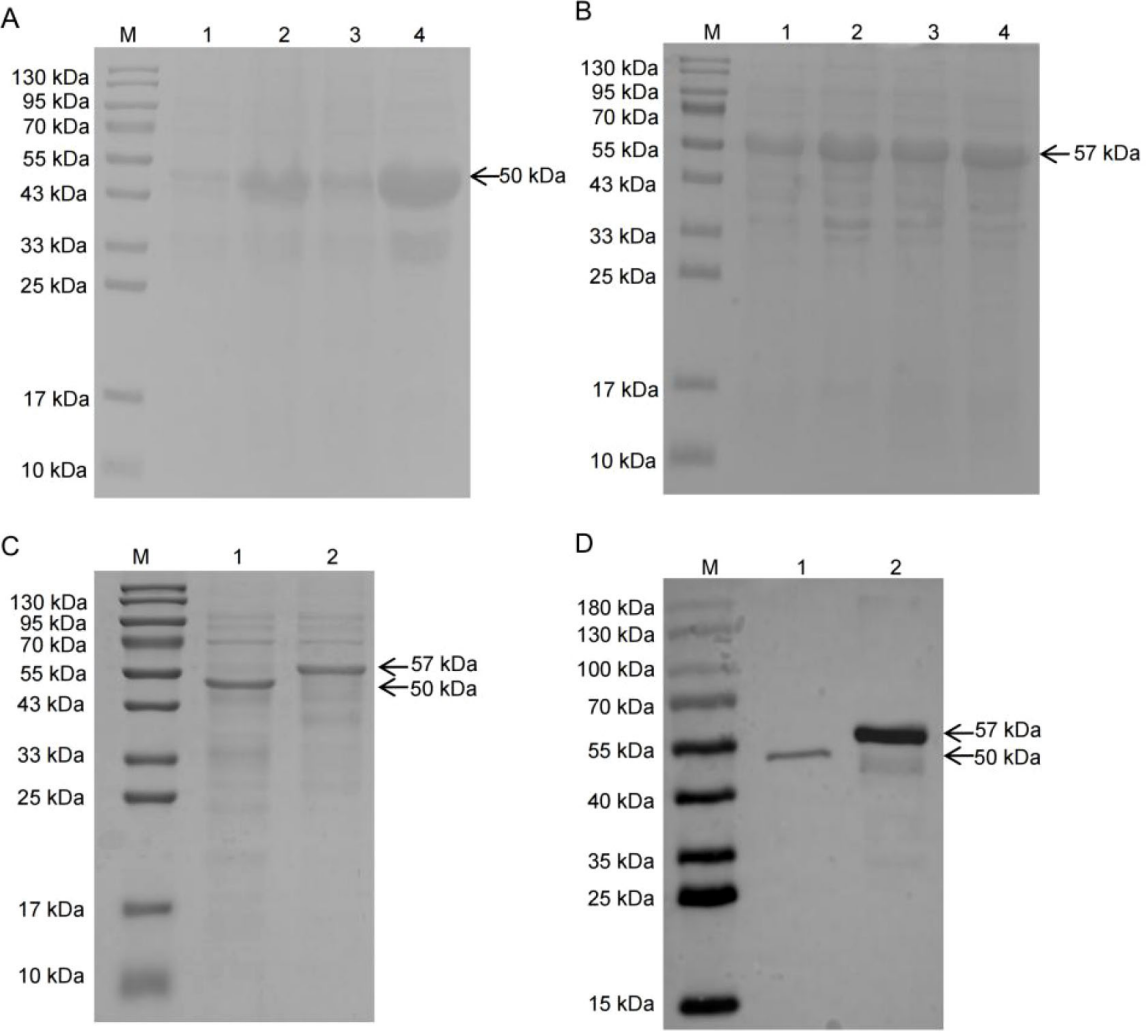
Expression, purification, and characterization of DBoV-A2 and DBoV-A4 fusion proteins. **(A)** SDS–PAGE analysis of DBoV-A2 expression. Lane M: protein molecular weight marker; lane 1: total bacterial lysate; lane 2: soluble supernatant fraction; lane 3: insoluble pellet fraction; lanes 4 and 5: samples before and after IPTG induction, respectively. **(B)** SDS–PAGE analysis of DBoV-A4 expression following the same layout as in **(A, C)** Purified fusion proteins after dialysis and ultrafiltration. **(D)** Western blot analysis using an anti-6×His tag antibody (AF2870; Beyotime, China) confirming the identity of DBoV-A2 and DBoV-A4 at their expected molecular weights.

The recombinant plasmid was verified through double digestion with NdeI and HindIII, and sequencing confirmed the intended nucleotide sequence. Following IPTG induction, SDS–PAGE revealed bands corresponding to the DBoV-A2 and DBoV-A4 fusion proteins at their anticipated molecular weights (**Figures 8A, B**). After purification via nickel affinity chromatography, dialysis, and ultrafiltration, SDS–PAGE confirmed that the proteins migrated to the expected sizes (**Figure 8C**). Western blot analysis was used to quantify the final protein concentration at 1 mg/mL (**Figure 8D**).

### DBoV-A2 and DBoV-A4 are safe in mice

**Figure 9:**
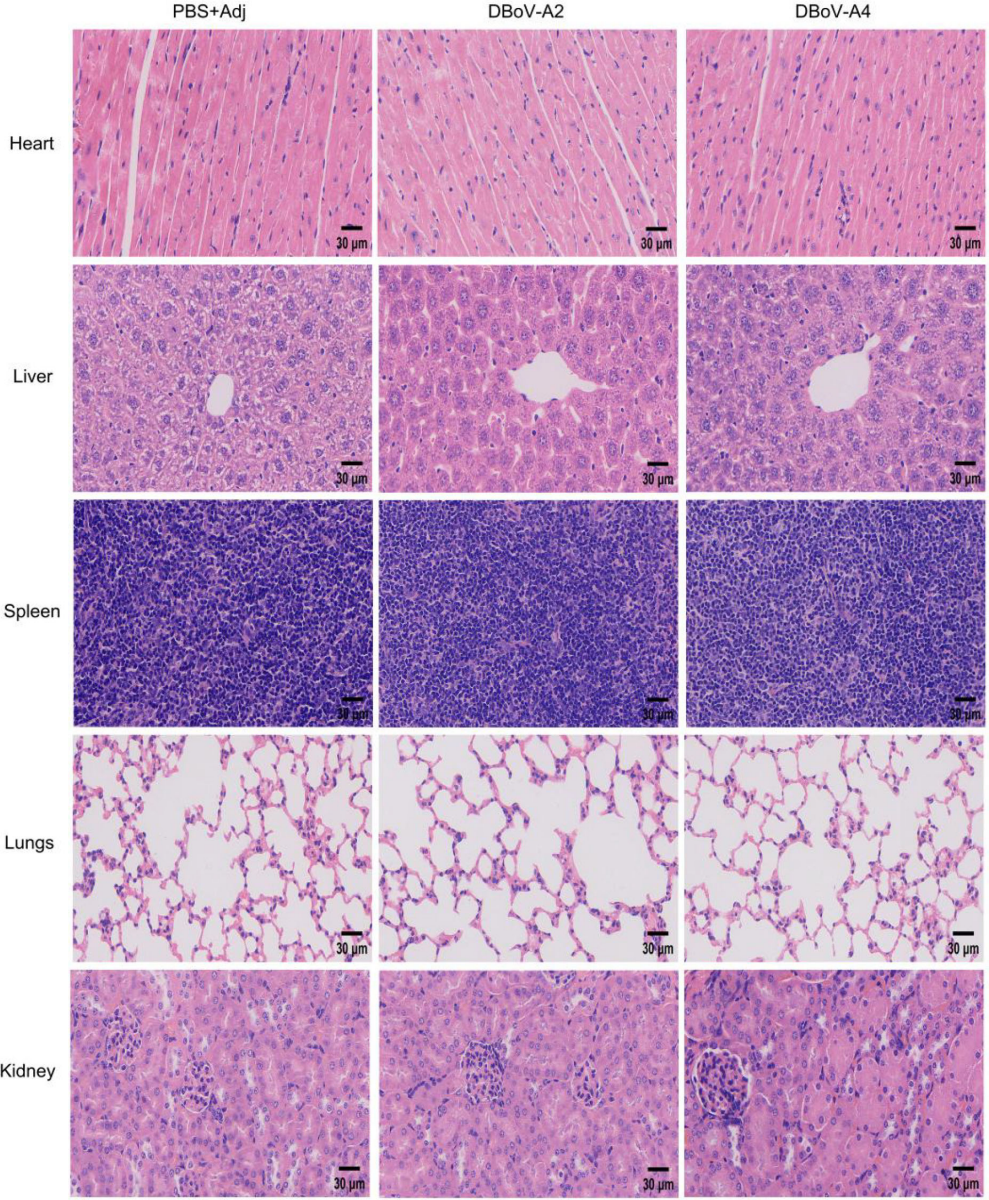
Initial vaccine safety assessment. After the full immunization schedule was completed, H&E staining was used to determine whether each antigen caused damage to the hearts, livers, spleens, lungs, and kidneys of the mice, thereby providing a preliminary evaluation of the safety and tolerance of the vaccine *in vivo* (scale bar, 30 μm).

While local reactions are an inherent feature of Freund’s adjuvant, the ultimate evaluation of any vaccine candidate must prioritize the systemic safety of the immunogen itself (48). To this end, two weeks post-immunization, heart, lung, liver, spleen, and kidney tissues were collected from BALB/c mice in each group for histological sectioning and hematoxylin and eosin (H&E) staining to assess whether the fusion proteins caused systemic organ damage.The results showed that, compared to the adjuvant control group, no significant pathological damage was observed in the major organs of mice immunized with the DBoV-A2 or DBoV-A4 fusion proteins. This finding indicates that both fusion proteins possess favorable systemic safety profiles, as they did not induce organic lesions in key organs such as the heart, lungs, liver, spleen, and kidneys. Any local reactions observed in the experiment (e.g., inflammation at the injection site) were confined to the area of adjuvant action and did not progress to systemic pathological changes. The DBoV-A2 and DBoV-A4 fusion proteins themselves exhibited no apparent organ toxicity and are therefore suitable for use as immunogens (**Figure 9**).

### DBoV-A2 and DBoV-A4 induce strong humoral and cellular immunity

**Figure 10:**
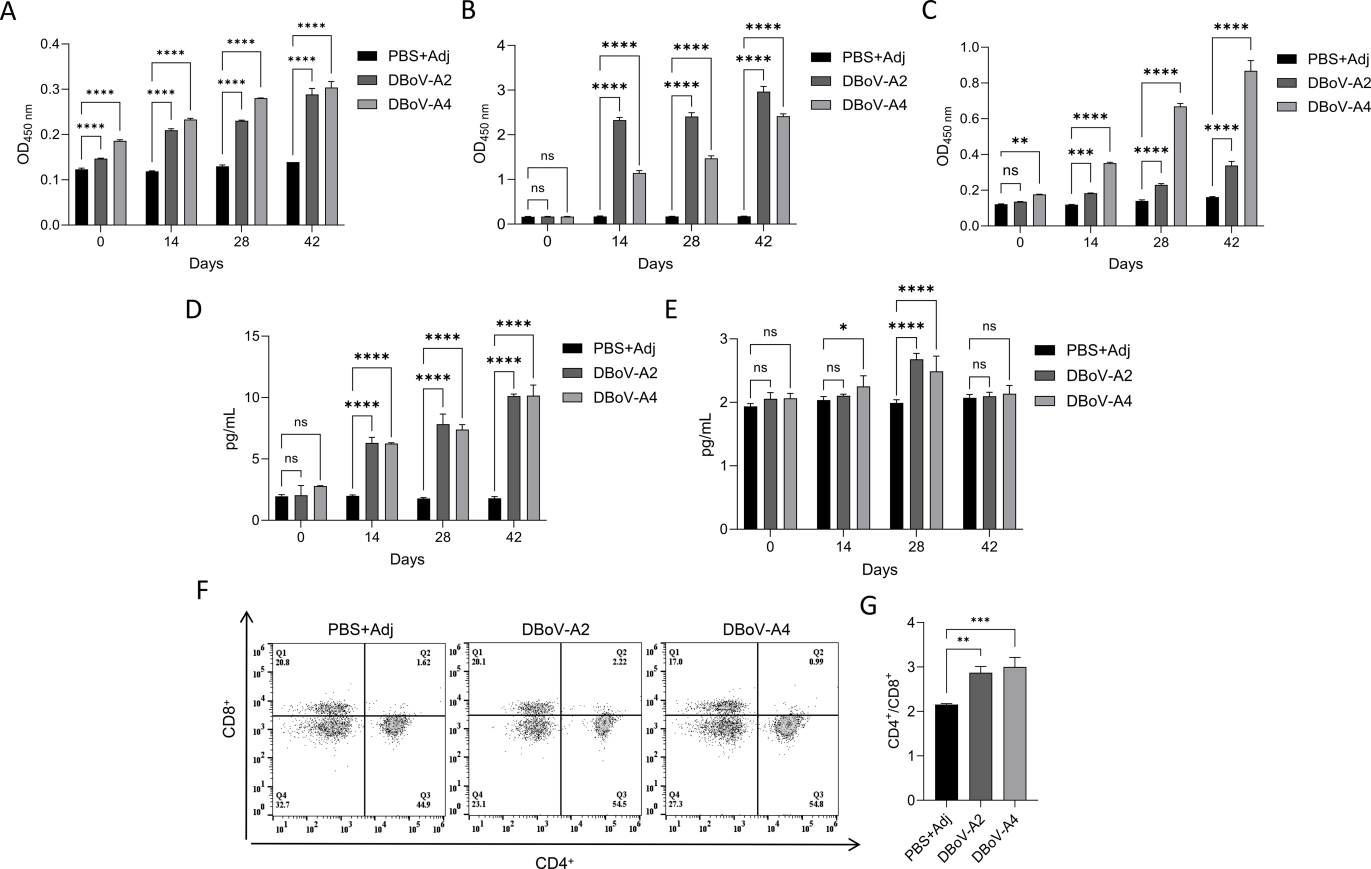
**(A–C)** Immune serum antibody titre analyses, where **** indicates statistically significant intergroup differences (*P* < 0.005). **(A)** Specific IgA antibody titres were determined using HRP-conjugated goat anti-mouse IgA (1:5,000 dilution) as the secondary antibody, with progressive titre elevation observed across immunizations. **(B)** IgG antibody titres were measured with HRP-conjugated goat anti-mouse IgG (1:5,000 dilution), which revealed similar immunization-dependent increases. **(C)** IgM antibody titre kinetics were determined via HRP-conjugated goat anti-mouse IgM (1:5,000 dilution), which exhibited comparable progressive enhancement with successive immunizations. **(D)** The expression levels of IL-4 in serum from the DBoV-A2 and DBoV-A4 groups were determined by enzyme-linked immunosorbent assay (ELISA). **(E)** IFN-γ expression levels in serum from the DBoV-A2 and DBoV-A4 groups were measured by ELISA. **(F)** The proportions of CD3^+^/CD4^+^ and CD3^+^CD8^+^ T lymphocytes were determined by flow cytometry. **(G)** Statistical analysis of the CD3^+^/CD4^+^/CD3^+^CD8^+^ ratio across different groups. Significance was calculated using an unpaired two-tailed Student’s t test (*P < 0.05; **P < 0.01; ***P < 0.001; ns indicates not statistically significant).

Serum samples were collected on day 7 after each immunization. The titers of DBoV-A2- and DBoV-A4-specific IgG, IgA, and IgM antibodies were measured by indirect ELISA. The results were as follows:IgA (**Figure 10A**): The titers increased in a stepwise manner with successive immunizations, peaking after the third immunization. The levels were significantly higher than those in the control group, indicating that IgA was the dominant antibody in the humoral immune response.IgG (**Figure 10B**): A significant increase was observed after the third immunization, suggesting the potential induction of a mucosal immune response.IgM (**Figure 10C**): A rapid increase was detected after the first immunization, followed by a plateau phase, which is consistent with the kinetics of an early primary response and subsequent class switching.Only a weak antibody response was observed in the adjuvant control group, confirming the specific immunogenicity of the fusion proteins. No significant differences were found between the DBoV-A2 and DBoV-A4 groups, indicating that both were equally effective in activating B cells and inducing a comprehensive humoral immune response.

To comprehensively evaluate the cellular immune responses induced by DBoV-A2 and DBoV-A4, serum cytokine levels and splenic T cell subsets were analyzed after the third immunization (**Figures 10D–G**). Cytokine Secretion (**Figures 10D, E**): Compared to the control group, serum levels of IFN-γ (a Th1 marker) and IL-4 (a Th2 marker) were significantly elevated in both experimental groups. This indicates that the vaccines successfully elicited a mixed Th1/Th2 immune response, which supports both antiviral cellular immunity and humoral immunity. In the flow cytometric analysis, a hierarchical gating strategy was employed to ensure the accuracy of the results. Initially, the lymphocyte population (P1) was identified from total splenocytes based on forward scatter (FSC) and side scatter (SSC) parameters. Subsequently, total T lymphocytes (P2) were gated from the P1 population using CD3^+^> staining. Finally, the cells within the P2 gate were analyzed for subpopulations by staining with CD4 and CD8 markers, respectively (**Figures 10F, G**; **Supplementary Table 7**): Flow cytometry results demonstrated that the proportion of CD4^+^> T cells and the CD4^+^>/CD8^+^> ratio were significantly higher in the experimental groups than in the control group, while the proportion of CD8^+^> T cells showed no significant change. These findings suggest that the immune response was predominantly characterized by the expansion of CD4^+^> helper T cells, providing support for B cell activation and antibody production.In summary, DBoV-A2 and DBoV-A4 effectively activated cellular immunity, primarily characterized by a CD4^+^> T cell response and accompanied by Th1/Th2-type cytokine secretion. Together with the humoral immunity data, these results constitute comprehensive evidence for a robust immune response.

## 4 Discussion

Studies have shown that porcine MHC alleles are phylogenetically closer to those of camelids (49). However, from the perspective of ruminants, camels and cattle both belong to the suborder Ruminantia, sharing numerous common characteristics in digestive physiology and immune system development. Consequently, their MHC molecules may retain a certain degree of structural conservation and functional similarity. Given the limited availability of camel MHC data, this study referred to the well-characterized MHC alleles of cattle—a closely related and extensively studied ruminant—as the immunogenetic background for epitope screening.

After establishing the MHC reference background for epitope screening, the next step was to identify the target proteins for the vaccine. Structural proteins represent the primary targets for developing epitope-based peptide vaccines (15). Paul et al. designed a novel multiepitope vaccine against canine parvovirus-2 (CPV-2) using immunoinformatic approaches (15). By utilizing the highly shared capsid protein VP2 for epitope screening, their vaccine demonstrated high antigenicity and immunogenicity and could elicit a robust immune response. Building upon this, in the present study, the VP1 and VP2 proteins of camel bocaparvovirus (DBoV) were selected to construct a multiepitope vaccine to increase its antigenicity and immunogenicity. Suitable B-cell epitopes, cytotoxic T-lymphocyte (CTL) epitopes, and helper T-lymphocyte (HTL) epitopes were screened and evaluated from these two structural proteins. The prediction of B-cell antigenic epitopes broadens the variety of antigenic peptides, thereby contributing to improved immunogenicity of the antigen (50). The objective of predicting HTL and CTL epitopes is to identify sequences within the antigen capable of stimulating CD4+ T cells or CD8+ T cells *in vivo (*20). In this study, 6 B-cell epitopes, 7 CTL epitopes, and 4 HTL epitopes were identified in the two proteins, and these epitopes were ultimately selected for vaccine construction.

To prevent the formation of neoepitopes, appropriate linkers were used during construction to connect the epitopes and adjuvants. Linkers are crucial for increasing protein stability and expression levels during multiepitope vaccine development. Three types of linkers can maintain proteins in an intrinsically ordered and typically folded state: flexible linkers, rigid linkers, and cleavable (or self-cleaving) linkers (51). In this study, flexible linkers were employed to ensure proper folding and functional independence of the fused domains: CTL epitopes were connected via AAY linkers, HTL epitopes via GPGPG linkers, and B−cell epitopes via KK linkers. These flexible linkers provide sufficient spatial freedom for each epitope to adopt its native conformation while preserving the overall structural integrity of the multi−epitope construct. Adjuvants have become key components of most vaccines and can increase cell-mediated immune responses, reduce the antigen dose, prolong the duration of the immune response, and function as agonists of Toll-like receptors (TLRs) (52). β-defensin activates APCs and NK cells through TLR1/2, thereby promoting adaptive immune responses and enhancing both cellular and humoral immunity (53). Heparin-binding haemagglutinin (HBHA) protein, an immunodominant antigen, is a TLR4 agonist can induce a Th1-type response, stimulate T cells, and promote IFN-γ production, thereby contributing to the development of effective immunotherapeutic strategies (54). Another adjuvant is the 50S ribosomal protein L7/L12, which improves immune function, particularly in dendritic cells that support innate and adaptive immunity and present antigens, and can recognize TLR4 to promote dendritic cell maturation (55). Flagellin (FliC) is a well-known TLR5 agonist that can effectively enhance antigen-specific serum IgG and IgA responses as well as mucosal IgA responses against various immunogens, thereby improving the effectiveness of vaccine construction (56). Concurrently, the Pan HLA-DR binding epitope (PADRE) sequence was introduced to increase vaccine stability and strengthen long-term immune responses through the induction of CD4+ T cells (57).

To validate the immunostimulatory capacity and sensitization risk of the vaccine constructs, this study systematically evaluated their antigenicity, immunogenicity, and allergenicity. The results indicated that all four constructs possessed antigenicity, immunogenicity, and non-allergenicity. This screening strategy is consistent with the computational validation workflows employed in multi-epitope vaccine studies for pathogens such as *Brucella* (58) and *Leptospira* (59). Predictions of physicochemical properties revealed that all four vaccine candidates exhibited favorable solubility and thermal stability. Secondary structure predictions showed that they adopted flexible and stable conformations conducive to antibody binding. A rational distribution of α-helices, extended strands, β-turns, and random coils provided a structural basis for the effective exposure of epitopes, a feature similar to the structural characteristics of the *Salmonella* Pullorum multi-epitope vaccine (60). Through multi-step prediction and optimization, the tertiary structures of the vaccines were elucidated. The final results demonstrated that DBoV-A2 and DBoV-A4 were superior to the other candidate vaccines. Furthermore, the prediction of B-cell conformational epitopes with high probability scores further confirmed the immunogenicity of the designed vaccines. This prediction methodology has been applied in studies of tuberculosis (61) and *Leptospira* vaccines (59).

TLR9 plays a central role in antiviral immunity by initiating the MyD88-dependent signaling pathway, promoting dendritic cell maturation, type I interferon production, and a Th1-biased adaptive immune response (62). Given the importance of TLR9 in antiviral defense, it was selected as a representative receptor in this study to evaluate the potential of DBoV-A2 and DBoV-A4 to activate innate immunity. This selection aligns with common practice in multi-epitope vaccine research, where TLR9 has been widely used as a target for molecular docking validation (63–66). Molecular docking results indicated that both vaccine constructs exhibited strong binding affinity to TLR9, which was further supported by subsequent molecular dynamics simulations confirming the stability of the complexes. These findings suggest that DBoV-A2 and DBoV-A4 may function as TLR9 agonists, potentially enhancing vaccine immunogenicity through direct triggering of innate immune signaling—an effect consistent with previous studies demonstrating that TLR9 activation promotes dendritic cell maturation and Th1 polarization (64). Similarly, the binding of DBoV−A2 and DBoV−A4 to TLR9 in this study can be interpreted as an immune−enhancing effect of the vaccine molecules in the host—by directly triggering innate immune signaling, they enhance vaccine immunogenicity and promote stronger induction of adaptive immune responses, rather than relying solely on conventional physical delivery functions. Molecular dynamics simulations further confirmed the high stability of this docking complex, which aligns with reported simulation results for multi−epitope vaccine–TLR9 complexes (63). Furthermore, immune simulations based on the vaccine sequences indicated that B−cell and T−cell populations gradually increased after three vaccine doses, peaking after the final administration. This dynamic trend is consistent with published immune simulation studies of multi−epitope vaccines (65–67). Concurrently, DBoV−A2 and DBoV−A4 elevated the levels of IFN−γ, TGF−β, IL−10, and IL−18. Among these, the increase in IFN−γ further corroborates a vaccine−induced Th1−biased response, which aligns with the role of TLR9 agonists in promoting Th1 polarization across various vaccine platforms (64–68). The elevated levels of immune cells and cytokines collectively demonstrate the excellent immunogenicity of DBoV−A2 and DBoV−A4. However, this computational finding has two major limitations. First, due to the lack of a camel TLR9 structure, bovine TLR9 was used as a surrogate, which may not fully capture species-specific interactions. Second, TLR9 is a CpG DNA receptor, and the observed protein-TLR9 binding does not equate to functional activation. Future TLR9 reporter assays are therefore necessary to validate the immunostimulatory potential of DBoV-A2 and DBoV-A4.

Based on preliminary immunoinformatics predictions, the DBoV-A2 and DBoV-A4 fusion proteins were successfully expressed and purified in this study. Histopathological evaluation demonstrated favorable systemic safety profiles for both proteins, with no significant pathological lesions observed in the major organs (heart, liver, spleen, lungs, and kidneys) of mice, confirming their *in vivo* biosafety. During the immunization process, all mice subjected to Freund’s Complete Adjuvant (FCA) exhibited mild to moderate local inflammatory reactions. Histological examination revealed inflammatory cell infiltration at the injection site, which is consistent with the known mechanism of Freund’s adjuvant—achieving sustained antigen release by inducing local recruitment of immune cells. Notably, no significant difference in the degree of local reactions was observed between the DBoV-A2/A4 immunization groups and the adjuvant control group, indicating that the observed local responses were primarily attributable to Freund’s adjuvant itself rather than to any toxic effects of the fusion proteins. These safety characteristics are consistent with the *in vivo* evaluation results of the *Toxoplasma gondii* multi-epitope vaccine USM.TOXO1 (69), further supporting the biosafety of DBoV-A2 and DBoV-A4 as candidate vaccines. In the immunogenicity evaluation, following three subcutaneous immunizations, serological analysis revealed that DBoV-A2 and DBoV-A4 significantly induced an increase in antigen-specific IgG, IgM, and IgA, confirming the elicitation of a humoral immune response. Similar high-level antibody responses have been reported in studies on Japanese encephalitis virus multi-epitope vaccines (70). Cytokine profile analysis indicated that both proteins induced substantial secretion of IL-4 and IFN-γ, suggesting the concurrent activation of both Th1 and Th2 immune pathways. This mixed response profile aligns with findings from the USM.TOXO1 study (69). In this study, IFN-γ exhibited a dynamic pattern of initial increase followed by a decline. Mechanistically, sustained Toll-like receptor 9 (TLR9) activation may induce IL-6 production, which in turn could suppress the continuous secretion of IFN-γ through immune feedback (71). Previous studies have confirmed that prolonged TLR9 stimulation significantly reduces the cytokine-secreting capacity of dendritic cells (62, 71), providing mechanistic support for the observed dynamic changes in IFN-γ in this study. Notably, no significant difference in IFN-γ levels was observed between the multi-epitope vaccine (MEV) group and the control group. This may be attributed to the potent immunostimulatory effect of Freund’s adjuvant itself, which elevated the baseline level in the control group, thereby partially masking the specific enhancement effect of the fusion proteins. Studies have confirmed that Freund’s adjuvant can significantly induce IFN-γ production and influence Th1-type responses (72, 73). The increase in CD4+ T cells post-vaccination indicates enhanced antigen recognition capability. Recent studies have confirmed that recombinant zoster vaccines (74) and rabies vaccines (75) can induce the persistence of antigen-specific memory CD4+ T cells, supporting the T-cell response characteristics observed in this study and suggesting that DBoV-A2 and DBoV-A4 may induce long-term immune memory.

This study demonstrated in a BALB/c mouse model that the DBoV-A2 and DBoV-A4 fusion proteins possess good immunogenicity, significantly enhancing the immune response, stimulating lymphocyte proliferation, and promoting the secretion of IL-4 and IFN-γ. These findings are highly consistent with the predictions from immunoinformatics analyses.It should be noted that this research is at an early proof-of-concept stage. Although the constructs incorporated four adjuvant elements, Freund’s Complete Adjuvant (FCA) was additionally used in animal immunization. The purpose of employing this potent immunostimulatory adjuvant was to reliably evaluate the immunogenic potential of the fusion proteins under optimal conditions. Due to its propensity to cause severe local reactions, the use of FCA is restricted to laboratory research and is not suitable for commercial vaccines.

This study has a major limitation, namely the lack of a challenge protection experiment. Furthermore, the use of Freund’s adjuvant obscured the independent contribution of the constructed built-in adjuvant. It must be specifically noted that due to differences in major histocompatibility complex (MHC) background, immune kinetics, and detection systems, mouse antibody titers cannot be directly extrapolated to camels; therefore, the mouse data can only provide qualitative evidence of immunogenicity. Additionally, due to the lack of MHC data for camelids, epitope screening relied on bovine MHC (BoLA) alleles. Future studies will address these shortcomings through challenge experiments, evaluation of built-in adjuvant activity, epitope optimization based on camelid MHC, and camel-specific immunogenicity tests. Regarding translational applications, based on the confirmed Toll-like receptor 9 (TLR9) binding affinity, subsequent work will focus on the development of TLR9 agonists, nanoadjuvant delivery systems, and approved veterinary adjuvants.

## Data Availability

The original contributions presented in the study are included in the article/**Supplementary Material**. Further inquiries can be directed to the corresponding author.
