# Supplementary material for: Preparation of a multiepitope vaccine candidate for camel bocavirus and evaluation of its immunogenicity in a mouse model
Source: Front Immunol. 2026 May 11;17:1786028. doi: 10.3389/fimmu.2026.1786028 (PMC13199348; doi:10.3389/fimmu.2026.1786028)
Supplement: Supplementary file 1 [file Table1.docx]

Supplementary Material

# Supplementary Data

Supplementary Material should be uploaded separately on submission. Please include any supplementary data, figures and/or tables.

Supplementary material is not typeset so please ensure that all information is clearly presented, the appropriate caption is included in the file and not in the manuscript, and that the style conforms to the rest of the article.

# Supplementary Figures and Tables

For more information on Supplementary Material and for details on the different file types accepted, please see [here](https://www.frontiersin.org/guidelines/author-guidelines#supplementary-material).

## Supplementary Figures

| Table S1 DBoV strain VP1 and VP2 protein sequence | | |
| --- | --- | --- |
| Protein | Serial number | Antigenicity |
| VP1 | YP_010796361.1 | 0.5206 |
| VP1 | YP_009389294.1 | 0.5446 |
| VP1 | ASC49364.1 | 0.5565 |
| VP1 | ASC49360.1 | 0.5269 |
| VP1 | ASC49284.1 | 0.5084 |
| VP1 | ASC49288.1 | 0.5612 |
| VP1 | ASC49296.1 | 0.5204 |
| VP1 | ASC49292.1 | 0.5127 |
| VP1 | ASC49300.1 | 0.5446 |
| VP1 | ASC49304.1 | 0.5491 |
| VP1 | ASC49308.1 | 0.4995 |
| VP1 | ASC49312.1 | 0.5814 |
| VP1 | ASC49316.1 | 0.5516 |
| VP1 | ASC49320.1 | 0.5337 |
| VP1 | ASC49324.1 | 0.5269 |
| VP1 | ASC49328.1 | 0.5219 |
| VP1 | ASC49332.1 | 0.5668 |
| VP1 | ASC49336.1 | 0.5206 |
| VP1 | ASC49340.1 | 0.5407 |
| VP1 | ASC49344.1 | 0.5425 |
| VP1 | ASC49348.1 | 0.5468 |
| VP1 | ASC49352.1 | 0.573 |
| VP1 | ASC49356.1 | 0.5505 |
| VP2 | YP_010796362.1 | 0.5075 |
| VP2 | YP_009389295.1 | 0.5433 |
| VP2 | ASC49365.1 | 0.5612 |
| VP2 | ASC49361.1 | 0.5241 |
| VP2 | ASC49357.1 | 0.5627 |
| VP2 | ASC49353.1 | 0.5837 |
| VP2 | ASC49349.1 | 0.5413 |
| VP2 | ASC49345.1 | 0.5461 |
| VP2 | ASC49341.1 | 0.54 |
| VP2 | ASC49337.1 | 0.5075 |
| VP2 | ASC49333.1 | 0.5667 |
| VP2 | ASC49329.1 | 0.5201 |
| VP2 | ASC49325.1 | 0.5174 |
| VP2 | ASC49321.1 | 0.533 |
| VP2 | ASC49317.1 | 0.5565 |
| VP2 | ASC49313.1 | 0.5908 |
| VP2 | ASC49309.1 | 0.4908 |
| VP2 | ASC49305.1 | 0.5489 |
| VP2 | ASC49301.1 | 0.5433 |
| VP2 | ASC49297.1 | 0.5091 |
| VP2 | ASC49293.1 | 0.5071 |
| VP2 | ASC49289.1 | 0.5688 |
| VP2 | ASC49285.1 | 0.5022 |

Table S2 B cell epitope screening

| Protein | Serial Number | Peptide | Antigenicity | Allergenicity | Toxicity | Mutagenicity |
| --- | --- | --- | --- | --- | --- | --- |
| VP1 | 1 | GGGIITPNRFVTRNTR | 0.4781 | Yes | No | No |
|  | 2 | EIEWEYETHFNKNWRP | 1.276 | No | No | No |
|  | 3 | QKTGYWKGGPGKTRNQ | 0.518 | No | No | No |
|  | 4 | LNPPTDKPTSNPDNPN | -0.272 | Yes | No | No |
|  | 5 | YGFSTPCPYFNFNQYN | 0.6748 | Yes | No | No |
|  | 6 | DSINITRYNPIWVKTP | 0.6464 | No | No | No |
|  | 7 | SGSSGNAFEEDYLAQE | 0.0732 | No | No | No |
|  | 8 | PGTIYLKLANIPVPST | 0.6417 | Yes | No | No |
|  | 9 | YEAIDDSGSSGNAFEE | 0.0138 | No | No | No |
|  | 10 | YKRYDAGTGARFYGFS | 0.6513 | Yes | No | No |
|  | 11 | ISSEYSYYEQQNMNQS | 0.7499 | No | No | No |
|  | 12 | EAAKRHDLAYNQYLNK | 0.2746 | No | No | No |
|  | 13 | KQYAYITCPYEAIDDS | 0.4184 | Yes | No | No |
|  | 14 | HAFPYTQNPWDTGTMP | 0.6279 | Yes | No | No |
|  | 15 | PVPSTHPESYLNVYAT | 0.2386 | No | No | No |
|  | 16 | QLSMQNPVWMMPNQAW | 0.4582 | Yes | No | No |
|  | 17 | GLHIFCDGEHAFPYTQ | 0.216 | No | No | No |
|  | 18 | QWWCDIKNEHKYKRYD | 0.7475 | No | No | No |
|  | 19 | VKRALAPSLNEKQLAP | 1.0395 | No | No | No |
|  | 20 | TSENIMRNAVYKVDNQ | -0.2839 | No | No | No |
|  | 21 | GTLPMSHPPGTIYLKL | 0.3571 | No | No | No |
|  | 22 | PSTDHAMPIHPAGTGT | 0.7609 | No | No | No |
|  | 23 | DKVWFCPKDHPSTDHA | 0.1537 | No | No | No |
|  | 24 | PELPTEPWELKQYAYI | 0.3698 | No | No | No |
|  | 25 | FVTRNTRQWWCDIKNE | 0.7512 | No | Yes | No |
|  | 26 | PQEAAAGSRAGAGGGP | 0.6602 | Yes | No | No |
|  | 27 | IWVKTPRVDRMTMVDT | -0.1308 | No | No | No |
|  | 28 | ANAGTGGVGMSTGQWI | 0.7694 | No | No | No |
|  | 29 | AEQKQARKLYFARSNK | 0.3461 | No | No | No |
|  | 30 | QGMYQWPINQQETMST | 0.2686 | No | No | No |
|  | 31 | IVRTGEAVEFHHEFNC | 1.1563 | Yes | No | No |
|  | 32 | DYLAQEPFYMLEQADH | 0.5564 | No | No | No |
|  | 33 | WDTGTMPELPTEPWEL | 0.0914 | No | No | No |
|  | 34 | YLGPFNPLDNGEPVNK | 0.9002 | No | No | No |
|  | 35 | PIHPAGTGTRNNLTFF | 1.1042 | No | No | No |
|  | 36 | SKECFRVTYGGADHNI | 1.0115 | No | No | No |
|  | 37 | YFARSNKGAKRQRLNP | 0.9488 | No | No | No |
|  | 38 | VGPPTGALAAMNEKDK | 0.7584 | No | No | No |
|  | 39 | QAKIETGIAVGPPTGA | 0.4273 | No | No | No |
|  | 40 | HEFNCGWVDNTRSAQT | 1.1391 | Yes | No | No |
|  | 41 | LFPGYNYLGPFNPLDN | 0.4333 | No | No | No |
|  | 42 | LGGKFARGVFSVKRAL | 0.634 | No | No | No |
|  | 43 | MEEEAAPQEAAAGSRA | 0.6697 | No | No | No |
|  | 44 | NPNMEAEQPMEEEAAP | 0.4705 | No | No | No |
|  | 45 | MPPAKQRGQFRGILFP | 0.6849 | Yes | No | No |
|  | 46 | TGTRNNLTFFTLENGQ | 0.7645 | Yes | No | No |
|  | 47 | TALGPNSSTDYAISSE | 0.6917 | Yes | No | No |
|  | 48 | VGMSTGQWIGGGIITP | 0.3958 | Yes | No | No |
|  | 49 | PSLNEKQLAPGRSAEQ | 1.1364 | No | No | No |
|  | 50 | RVDRMTMVDTRDGTLP | 0.9053 | Yes | No | No |
|  | 51 | YGGADHNILAKRNQKT | 0.6329 | No | No | No |
|  | 52 | ASMSMNPEILSKECFR | 1.0012 | Yes | No | No |
|  | 53 | TRSAQTPASMSMNPEI | 0.9211 | No | No | No |
|  | 54 | GEPVNKADEAAKRHDL | 0.7504 | Yes | No | No |
|  | 55 | PKEWQTLLNIAKRFRP | 0.4624 | No | No | No |
|  | 56 | PVWMMPNQAWDSINIT | 0.7658 | No | No | No |
|  | 57 | FYMLEQADHMIVRTGE | 0.6526 | Yes | No | No |
|  | 58 | NKGLNPYLKFNKADQQ | 0.6693 | No | No | No |
|  | 59 | GGPGKTRNQTALGPNS | 1.0955 | No | No | No |
|  | 60 | KPTSNPDNPNMEAEQP | 0.331 | No | No | No |
|  | 61 | PINQQETMSTRYGMEK | 0.424 | Yes | No | No |
|  | 62 | NIQIKQIVTNAANETL | 0.366 | No | No | No |
|  | 63 | IAKRFRPVRMTVKVYN | 0.6881 | No | No | No |
|  | 64 | YNGHFSPKEWQTLLNI | 1.0422 | No | No | No |
|  | 65 | DQQLIDDLSNDSSLGG | 0.2495 | No | No | No |
|  | 66 | YEQQNMNQSGDQAKIE | 0.4453 | No | No | No |
|  | 67 | SYLNVYATGNVSVEIE | 0.7535 | No | No | No |
| VP2 | 68 | GGGIITPNRFVTRNTR | 0.4781 | Yes | No | No |
|  | 69 | EIEWEYETHFNKNWRP | 1.276 | No | No | No |
|  | 70 | QKTGYWKGGPGKTRNQ | 0.518 | No | No | No |
|  | 71 | YGFSTPCPYFNFNQYN | 0.6748 | Yes | No | No |
|  | 72 | DSINITRYNPIWVKTP | 0.6464 | No | No | No |
|  | 73 | SGSSGNAFEEDYLAQE | 0.0732 | No | No | No |
|  | 74 | YKRYDAGTGARFYGFS | 0.6513 | Yes | No | No |
|  | 75 | PGTIYLKLANIPVPST | 0.6417 | Yes | No | No |
|  | 76 | YEAIDDSGSSGNAFEE | 0.0138 | No | No | No |
|  | 77 | ISSEYSYYEQQNMNQS | 0.7499 | No | No | No |
|  | 78 | KQYAYITCPYEAIDDS | 0.4184 | Yes | No | No |
|  | 79 | HAFPYTQNPWDTGTMP | 0.6279 | Yes | No | No |
|  | 80 | QWWCDIKNEHKYKRYD | 0.7475 | No | Yes | No |
|  | 81 | PVPSTHPESYLNVYAT | 0.2386 | No | No | No |
|  | 82 | QLSMQNPVWMMPNQAW | 0.4582 | Yes | No | No |
|  | 83 | GLHIFCDGEHAFPYTQ | 0.216 | No | No | No |
|  | 84 | FVTRNTRQWWCDIKNE | 0.7512 | No | Yes | No |
|  | 85 | TSENIMRNAVYKVDNQ | -0.2839 | No | No | No |
|  | 86 | GTLPMSHPPGTIYLKL | 0.3571 | No | No | No |
|  | 87 | PSTDHAMPIHPAGTGT | 0.7609 | No | No | No |
|  | 88 | DKVWFCPKDHPSTDHA | 0.1537 | No | No | No |
|  | 89 | PELPTEPWELKQYAYI | 0.3698 | No | No | No |
|  | 90 | PQEAAAGSRAGAGGGP | 0.6602 | Yes | No | No |
|  | 91 | IWVKTPRVDRMTMVDT | -0.1308 | No | No | No |
|  | 92 | ANAGTGGVGMSTGQWI | 0.7694 | No | No | No |
|  | 93 | QGMYQWPINQQETMST | 0.2686 | No | No | No |
|  | 94 | IVRTGEAVEFHHEFNC | 1.1563 | Yes | No | No |
|  | 95 | DYLAQEPFYMLEQADH | 0.5564 | No | No | No |
|  | 96 | WDTGTMPELPTEPWEL | 0.0914 | No | No | No |
|  | 97 | PIHPAGTGTRNNLTFF | 1.1042 | No | No | No |
|  | 98 | SKECFRVTYGGADHNI | 1.0115 | No | No | No |
|  | 99 | VGPPTGALAAMNEKDK | 0.7584 | No | No | No |
|  | 100 | QAKIETGIAVGPPTGA | 0.4273 | No | No | No |
|  | 101 | HEFNCGWVDNTRSAQT | 1.1391 | Yes | No | No |
|  | 102 | MEEEAAPQEAAAGSRA | 0.6697 | No | No | No |
|  | 103 | TGTRNNLTFFTLENGQ | 0.7645 | Yes | No | No |
|  | 104 | VGMSTGQWIGGGIITP | 0.3958 | No | No | No |
|  | 105 | TALGPNSSTDYAISSE | 0.6917 | Yes | No | No |
|  | 106 | RVDRMTMVDTRDGTLP | 0.9053 | Yes | No | No |
|  | 107 | YGGADHNILAKRNQKT | 0.6329 | No | No | No |
|  | 108 | ASMSMNPEILSKECFR | 1.0012 | Yes | No | No |
|  | 109 | TRSAQTPASMSMNPEI | 0.9211 | No | No | No |
|  | 110 | PKEWQTLLNIAKRFRP | 0.4624 | No | No | No |
|  | 111 | PVWMMPNQAWDSINIT | 0.7658 | No | No | No |
|  | 112 | FYMLEQADHMIVRTGE | 0.6526 | Yes | No | No |
|  | 113 | GGPGKTRNQTALGPNS | 1.0955 | No | No | No |
|  | 114 | PINQQETMSTRYGMEK | 0.424 | Yes | No | No |
|  | 115 | NIQIKQIVTNAANETL | 0.366 | No | No | No |
|  | 116 | YNGHFSPKEWQTLLNI | 1.0422 | No | No | No |
|  | 117 | IAKRFRPVRMTVKVYN | 0.6881 | No | No | No |
|  | 118 | YEQQNMNQSGDQAKIE | 0.4453 | No | No | No |
|  | 119 | SYLNVYATGNVSVEIE | 0.7535 | No | No | No |

Table S3 CTL epitope screening

| Protein | Allele | Serial Number | Peptide | Antigenicity | Immunogenicity | Allergenicity | Toxicity | Mutagenicity |
| --- | --- | --- | --- | --- | --- | --- | --- | --- |
| VP1 | BoLA-HD6 | 3 | YLKFNKADQQL | 0.5574 | -0.27496 | No | No | No |
|  |  | 9 | GVFSVKRAL | 1.0037 | -0.22473 | No | No | No |
|  | BoLA-JSP.1 | 12 | GHFSPKEWQTL | 1.5201 | -0.11834 | No | No | No |
|  |  | 14 | YNYLGPFNPL | 0.6946 | 0.1081 | No | No | No |
|  |  | 15 | LAPSLNEKQL | 1.348 | -0.3426 | No | No | No |
|  |  | 16 | HFSPKEWQTL | 1.4092 | -0.04393 | No | No | No |
|  |  | 17 | FSPKEWQTLL | 0.7867 | 0.00984 | No | No | No |
|  |  | 20 | FSPKEWQTL | 1.2179 | 0.01033 | No | No | No |
|  |  | 21 | FQNDLTAGL | 0.616 | 0.09878 | No | No | No |
|  |  | 22 | RNNLTFFTL | 0.7767 | 0.25622 | No | No | No |
|  | BoLA-T2c | 27 | GVFSVKRAL | 1.0037 | -0.22473 | No | No | No |
|  |  | 29 | FQNDLTAGL | 0.616 | 0.09878 | No | No | No |
|  | BoLA-T2b | 36 | AEQKQARKLYF | 0.5054 | -0.49342 | No | No | No |
|  |  | 37 | SEYSYYEQQNM | 0.888 | -0.28795 | No | No | No |
|  | BoLA-T2a | 45 | KRALAPSLNEK | 1.1826 | -0.08966 | No | No | No |
|  |  | 47 | RALAPSLNEK | 1.1697 | -0.09353 | No | No | No |
|  |  | 49 | MSMNPEILSK | 1.2433 | 0.04245 | No | No | No |
|  |  | 51 | TGALAAMNEK | 1.0644 | -0.03452 | No | No | No |
|  |  | 53 | SSLGGKFAR | 1.0601 | -0.01784 | No | No | No |
|  |  | 54 | SMNPEILSK | 1.2558 | 0.1035 | No | No | No |
|  |  | 60 | KVYNIQIK | 1.2557 | 0.12517 | No | No | No |
|  | BoLA-T7 | 63 | FQNDLTAGL | 0.616 | 0.09878 | No | No | No |
|  |  | 65 | RSAQTPASM | 0.585 | -0.14014 | No | No | No |
|  |  | 66 | YSYYEQQNM | 0.853 | -0.118 | No | No | No |
|  |  | 68 | MVDTRDGTL | 1.5191 | 0.16882 | No | No | No |
|  |  | 69 | TIYLKLANI | 1.2999 | -0.20356 | No | No | No |
|  | BoLA-D18.4 | 72 | VKRALAPSL | 0.5176 | -0.02382 | No | No | No |
|  |  | 73 | KQLAPGRSA | 0.8431 | 0.00389 | No | No | No |
|  |  | 75 | QKQARKLYF | 0.5628 | -0.16235 | No | No | No |
|  |  | 77 | FQNDLTAGL | 0.616 | 0.09878 | No | No | No |
|  |  | 78 | AQTPASMSM | 0.7167 | -0.36105 | No | No | No |
|  |  | 81 | GKTRNQTAL | 0.9701 | 0.00496 | No | No | No |
|  |  | 82 | AKIETGIAV | 0.5349 | 0.34883 | No | No | No |
|  |  | 86 | YKVDNQGMY | 0.4906 | -0.15362 | No | No | No |
|  |  | 90 | KQARKLYF | 0.7744 | -0.15878 | No | No | No |
|  | BoLA-AW10 | 94 | LAPSLNEKQL | 1.348 | -0.3426 | No | No | No |
|  |  | 95 | HFSPKEWQTL | 1.4092 | -0.04393 | No | No | No |
|  |  | 96 | FSPKEWQTLL | 0.7867 | 0.00984 | No | No | No |
|  |  | 100 | FSPKEWQTL | 1.2179 | 0.01033 | No | No | No |
|  |  | 104 | RNNLTFFTL | 0.7767 | 0.25622 | No | No | No |
|  | BoLA-T5 | 110 | FQNDLTAGLH | 0.5695 | 0.10617 | No | No | No |
|  |  | 111 | KQYAYITCPY | 0.7575 | 0.14873 | No | No | No |
|  |  | 114 | KQLAPGRSA | 0.8431 | 0.00389 | No | No | No |
|  |  | 116 | AQTPASMSM | 0.7167 | -0.36105 | No | No | No |
|  |  | 120 | AKIETGIAV | 0.5349 | 0.34883 | No | No | No |
|  |  | 123 | YKVDNQGMY | 0.4906 | -0.15362 | No | No | No |
|  |  | 124 | QQETMSTRY | 0.6325 | -0.19217 | No | No | No |
|  |  | 127 | KQARKLYF | 0.7744 | -0.15878 | No | No | No |
| VP2 | BoLA-JSP.1 | 6 | FSPKEWQTLL | 0.7867 | 0.00984 | No | No | No |
|  |  | 9 | FSPKEWQTL | 1.2179 | 0.01033 | No | No | No |
|  |  | 10 | FQNDLTAGL | 0.616 | 0.09878 | No | No | No |
|  |  | 11 | RNNLTFFTL | 0.7767 | 0.25622 | No | No | No |
|  | BoLA-T2c | 16 | FQNDLTAGL | 0.616 | 0.09878 | No | No | No |
|  | BoLA-T2a | 27 | MSMNPEILSK | 1.2433 | 0.04245 | No | No | No |
|  |  | 30 | SMNPEILSK | 1.2558 | 0.1035 | No | No | No |
|  |  | 35 | KVYNIQIK | 1.2557 | 0.12517 | No | No | No |
|  | BoLA-T7 | 37 | FQNDLTAGL | 0.616 | 0.09878 | No | No | No |
|  |  | 42 | MVDTRDGTL | 1.5191 | 0.16882 | No | No | No |
|  | BoLA-D18.4 | 46 | FQNDLTAGL | 0.616 | 0.09878 | No | No | No |
|  |  | 50 | GKTRNQTAL | 0.9701 | 0.00496 | No | No | No |
|  |  | 51 | AKIETGIAV | 0.5349 | 0.34883 | No | No | No |
|  | BoLA-AW10 | 59 | FSPKEWQTLL | 0.7867 | 0.00984 | No | No | No |
|  |  | 63 | FSPKEWQTL | 1.2179 | 0.01033 | No | No | No |
|  |  | 67 | RNNLTFFTL | 0.7767 | 0.25622 | No | No | No |
|  | BoLA-T5 | 73 | FQNDLTAGLH | 0.5695 | 0.10617 | No | No | No |
|  |  | 74 | KQYAYITCPY | 0.7575 | 0.14873 | No | No | No |
|  |  | 79 | AKIETGIAV | 0.5349 | 0.34883 | No | No | No |

Table S4 HTL epitope screening

| Protein | Allele | Serial Number | Peptide | Antigenicity | Allergenicity | Toxicity | Mutagenicity | IFN | IL-4 |
| --- | --- | --- | --- | --- | --- | --- | --- | --- | --- |
| VP1 | BoLA-DRB3*0101 | 1 | FARGVFSVKRALAPS | 0.717 | No | No | No | Yes | No |
|  |  | 2 | ARGVFSVKRALAPSL | 0.8955 | No | No | No | Yes | No |
|  |  | 3 | RGVFSVKRALAPSLN | 0.9039 | No | No | No | Yes | No |
|  |  | 4 | GVFSVKRALAPSLNE | 0.8374 | No | No | No | Yes | No |
|  |  | 5 | VFSVKRALAPSLNEK | 1.0127 | No | No | No | Yes | No |
|  |  | 6 | FSVKRALAPSLNEKQ | 1.0596 | No | No | No | Yes | No |
|  |  | 7 | KLYFARSNKGAKRQR | 0.4097 | No | No | No | Yes | Yes |
|  |  | 8 | EWQTLLNIAKRFRPV | 0.7848 | No | No | No | Yes | Yes |
|  |  | 9 | QTLLNIAKRFRPVRM | 0.4959 | No | No | No | Yes | No |
|  |  | 10 | TLLNIAKRFRPVRMT | 0.8397 | No | No | No | Yes | No |
|  |  | 11 | LLNIAKRFRPVRMTV | 0.6578 | No | No | No | Yes | No |
|  |  | 12 | LNIAKRFRPVRMTVK | 0.911 | No | No | No | Yes | No |
|  |  | 13 | NIAKRFRPVRMTVKV | 0.6446 | No | No | No | Yes | No |
|  |  | 14 | IAKRFRPVRMTVKVY | 0.5942 | No | No | No | Yes | Yes |
|  |  | 15 | AKRFRPVRMTVKVYN | 0.6025 | Yes | No | No | Yes | No |
|  |  | 16 | KRFRPVRMTVKVYNI | 0.7767 | No | No | No | Yes | Yes |
|  |  | 17 | RFRPVRMTVKVYNIQ | 0.9837 | Yes | No | No | Yes | No |
|  |  | 18 | FRPVRMTVKVYNIQI | 1.1223 | Yes | No | No | Yes | No |
|  |  | 19 | RPVRMTVKVYNIQIK | 1.1861 | Yes | No | No | Yes | No |
|  |  | 20 | WDSINITRYNPIWVK | 1.1609 | Yes | No | No | Yes | No |
|  |  | 21 | DSINITRYNPIWVKT | 0.9188 | No | No | No | Yes | Yes |
|  |  | 22 | SINITRYNPIWVKTP | 0.7839 | No | No | No | Yes | Yes |
|  |  | 23 | INITRYNPIWVKTPR | 0.6575 | Yes | No | No | Yes | No |
|  |  | 24 | AYNQYLNKGLNPYLK | 0.469 | No | No | No | Yes | Yes |
|  |  | 25 | LGGKFARGVFSVKRA | 0.8654 | No | No | No | Yes | No |
|  |  | 26 | GGKFARGVFSVKRAL | 0.4456 | Yes | No | No | Yes | No |
|  |  | 27 | FARGVFSVKRALAPS | 0.717 | No | No | No | Yes | No |
|  |  | 28 | ARGVFSVKRALAPSL | 0.8955 | No | No | No | Yes | No |
|  |  | 29 | RGVFSVKRALAPSLN | 0.9039 | No | No | No | Yes | No |
|  |  | 30 | GVFSVKRALAPSLNE | 0.8374 | No | No | No | Yes | No |
|  |  | 31 | VFSVKRALAPSLNEK | 1.0127 | No | No | No | Yes | No |
|  |  | 32 | FSVKRALAPSLNEKQ | 1.0596 | No | No | No | Yes | No |
|  |  | 33 | KLYFARSNKGAKRQR | 0.4097 | No | No | No | Yes | Yes |
|  |  | 34 | EWQTLLNIAKRFRPV | 0.7848 | No | No | No | Yes | Yes |
|  |  | 35 | QTLLNIAKRFRPVRM | 0.4959 | No | No | No | Yes | No |
|  |  | 36 | TLLNIAKRFRPVRMT | 0.8397 | No | No | No | Yes | No |
|  |  | 37 | LLNIAKRFRPVRMTV | 0.6578 | No | No | No | Yes | No |
|  |  | 38 | LNIAKRFRPVRMTVK | 0.911 | No | No | No | Yes | No |
|  |  | 39 | NIAKRFRPVRMTVKV | 0.6446 | No | No | No | Yes | No |
|  |  | 40 | IAKRFRPVRMTVKVY | 0.5942 | No | No | No | Yes | Yes |
|  |  | 41 | AKRFRPVRMTVKVYN | 0.6025 | Yes | No | No | Yes | No |
|  |  | 42 | KRFRPVRMTVKVYNI | 0.7767 | No | No | No | Yes | Yes |
|  |  | 43 | AWDSINITRYNPIWV | 1.1852 | No | No | No | Yes | Yes |
|  |  | 44 | DSINITRYNPIWVKT | 0.9188 | No | No | No | Yes | Yes |
|  |  | 45 | SINITRYNPIWVKTP | 0.7839 | No | No | No | Yes | Yes |
|  |  | 46 | HPPGTIYLKLANIPV | 0.7353 | No | No | No | Yes | No |
|  | BoLA-DRB3_1501 | 47 | AWDSINITRYNPIWV | 1.1852 | No | No | No | Yes | Yes |
|  |  | 48 | DSINITRYNPIWVKT | 0.9188 | No | No | No | Yes | Yes |
|  |  | 49 | SINITRYNPIWVKTP | 0.7839 | No | No | No | Yes | Yes |
|  | BoLA-DRB3*14011 | 50 | FARGVFSVKRALAPS | 0.717 | No | No | No | Yes | No |
|  |  | 51 | ARGVFSVKRALAPSL | 0.8955 | No | No | No | Yes | No |
|  |  | 52 | RGVFSVKRALAPSLN | 0.9039 | No | No | No | Yes | No |
|  |  | 53 | GVFSVKRALAPSLNE | 0.8374 | No | No | No | Yes | No |
|  |  | 54 | VFSVKRALAPSLNEK | 1.0127 | No | No | No | Yes | No |
|  |  | 55 | FSVKRALAPSLNEKQ | 1.0596 | No | No | No | Yes | No |
|  |  | 56 | KLYFARSNKGAKRQR | 0.4097 | No | No | No | Yes | Yes |
|  |  | 57 | EWQTLLNIAKRFRPV | 0.7848 | No | No | No | Yes | Yes |
|  |  | 58 | QTLLNIAKRFRPVRM | 0.4959 | No | No | No | Yes | No |
|  |  | 59 | TLLNIAKRFRPVRMT | 0.8397 | No | No | No | Yes | No |
|  |  | 60 | LLNIAKRFRPVRMTV | 0.6578 | No | No | No | Yes | No |
|  |  | 61 | LNIAKRFRPVRMTVK | 0.911 | No | No | No | Yes | No |
|  |  | 62 | NIAKRFRPVRMTVKV | 0.6446 | No | No | No | Yes | No |
|  |  | 63 | IAKRFRPVRMTVKVY | 0.5942 | No | No | No | Yes | Yes |
|  |  | 64 | KRFRPVRMTVKVYNI | 0.7767 | No | No | No | Yes | Yes |
|  |  | 65 | AWDSINITRYNPIWV | 1.1852 | No | No | No | Yes | Yes |
|  |  | 66 | DSINITRYNPIWVKT | 0.9188 | No | No | No | Yes | Yes |
|  |  | 67 | SINITRYNPIWVKTP | 0.7839 | No | No | No | Yes | Yes |
| VP2 | BoLA-DRB3*0101 | 68 | KEWQTLLNIAKRFRP | 0.4655 | No | No | No | No | No |
|  |  | 69 | EWQTLLNIAKRFRPV | 0.7848 | No | No | No | Yes | Yes |
|  |  | 70 | QTLLNIAKRFRPVRM | 0.4959 | No | No | No | Yes | No |
|  |  | 71 | TLLNIAKRFRPVRMT | 0.8397 | No | No | No | Yes | No |
|  |  | 72 | LLNIAKRFRPVRMTV | 0.6578 | No | No | No | Yes | No |
|  |  | 73 | LNIAKRFRPVRMTVK | 0.911 | No | No | No | Yes | No |
|  |  | 74 | NIAKRFRPVRMTVKV | 0.6446 | No | No | No | Yes | No |
|  |  | 75 | IAKRFRPVRMTVKVY | 0.5942 | No | No | No | Yes | Yes |
|  |  | 76 | KRFRPVRMTVKVYNI | 0.7767 | No | No | No | Yes | Yes |
|  |  | 77 | YNIQIKQIVTNAANE | 0.4031 | No | No | No | No | No |
|  |  | 78 | NIQIKQIVTNAANET | 0.4615 | No | No | No | No | No |
|  |  | 79 | ADHNILAKRNQKTGY | 1.0466 | No | No | No | No | No |
|  |  | 80 | DHNILAKRNQKTGYW | 1.1327 | No | No | No | No | No |
|  |  | 81 | HNILAKRNQKTGYWK | 0.7893 | No | No | No | No | No |
|  |  | 82 | NILAKRNQKTGYWKG | 0.7015 | No | No | No | No | No |
|  |  | 83 | DSINITRYNPIWVKT | 0.9188 | No | No | No | Yes | Yes |
|  |  | 84 | SINITRYNPIWVKTP | 0.7839 | No | No | No | Yes | Yes |
|  |  | 85 | TIYLKLANIPVPSTH | 0.6298 | No | No | No | Yes | No |
|  |  | 86 | KEWQTLLNIAKRFRP | 0.4655 | No | No | No | No | No |
|  |  | 87 | EWQTLLNIAKRFRPV | 0.7848 | No | No | No | Yes | Yes |
|  |  | 88 | QTLLNIAKRFRPVRM | 0.4959 | No | No | No | Yes | No |
|  |  | 89 | TLLNIAKRFRPVRMT | 0.8397 | No | No | No | Yes | No |
|  |  | 90 | LLNIAKRFRPVRMTV | 0.6578 | No | No | No | Yes | No |
|  |  | 91 | LNIAKRFRPVRMTVK | 0.911 | No | No | No | Yes | No |
|  |  | 92 | NIAKRFRPVRMTVKV | 0.6446 | No | No | No | Yes | No |
|  |  | 93 | IAKRFRPVRMTVKVY | 0.5942 | No | No | No | Yes | Yes |
|  |  | 94 | KRFRPVRMTVKVYNI | 0.7767 | No | No | No | Yes | Yes |
|  |  | 95 | YNIQIKQIVTNAANE | 0.4031 | No | No | No | No | No |
|  |  | 96 | NIQIKQIVTNAANET | 0.4615 | No | No | No | No | No |
|  |  | 97 | GGADHNILAKRNQKT | 0.6607 | No | No | No | No | No |
|  |  | 98 | GADHNILAKRNQKTG | 0.9074 | No | No | No | No | No |
|  |  | 99 | ADHNILAKRNQKTGY | 1.0466 | No | No | No | No | No |
|  |  | 100 | DHNILAKRNQKTGYW | 1.1327 | No | No | No | No | No |
|  |  | 101 | HNILAKRNQKTGYWK | 0.7893 | No | No | No | No | No |
|  |  | 102 | NILAKRNQKTGYWKG | 0.7015 | No | No | No | No | No |
|  |  | 103 | AWDSINITRYNPIWV | 1.1852 | No | No | No | Yes | Yes |
|  |  | 104 | DSINITRYNPIWVKT | 0.9188 | No | No | No | Yes | Yes |
|  |  | 105 | SINITRYNPIWVKTP | 0.7839 | No | No | No | Yes | Yes |
|  |  | 106 | SHPPGTIYLKLANIP | 0.75 | No | No | No | No | No |
|  |  | 107 | HPPGTIYLKLANIPV | 0.7353 | No | No | No | Yes | No |
|  |  | 108 | PPGTIYLKLANIPVP | 0.6052 | No | No | No | No | No |
|  |  | 109 | TIYLKLANIPVPSTH | 0.6298 | No | No | No | Yes | No |
|  | BoLA-DRB3*14011 | 110 | KEWQTLLNIAKRFRP | 0.4655 | No | No | No | No | No |
|  |  | 111 | EWQTLLNIAKRFRPV | 0.7848 | No | No | No | Yes | Yes |
|  |  | 112 | QTLLNIAKRFRPVRM | 0.4959 | No | No | No | Yes | No |
|  |  | 113 | TLLNIAKRFRPVRMT | 0.8397 | No | No | No | Yes | No |
|  |  | 114 | LLNIAKRFRPVRMTV | 0.6578 | No | No | No | Yes | No |
|  |  | 115 | LNIAKRFRPVRMTVK | 0.911 | No | No | No | Yes | No |
|  |  | 116 | NIAKRFRPVRMTVKV | 0.6446 | No | No | No | Yes | No |
|  |  | 117 | IAKRFRPVRMTVKVY | 0.5942 | No | No | No | Yes | Yes |
|  |  | 118 | KRFRPVRMTVKVYNI | 0.7767 | No | No | No | Yes | Yes |
|  |  | 119 | YNIQIKQIVTNAANE | 0.4031 | No | No | No | No | No |
|  |  | 120 | NIQIKQIVTNAANET | 0.4615 | No | No | No | No | No |
|  |  | 121 | AWDSINITRYNPIWV | 1.1852 | No | No | No | Yes | Yes |
|  |  | 122 | DSINITRYNPIWVKT | 0.9188 | No | No | No | Yes | Yes |
|  |  | 123 | SINITRYNPIWVKTP | 0.7839 | No | No | No | Yes | Yes |
|  |  | 124 | PPGTIYLKLANIPVP | 0.6052 | No | No | No | No | No |
|  |  | 125 | TIYLKLANIPVPSTH | 0.6298 | No | No | No | Yes | No |
|  | BoLA-DRB3*1501 | 126 | AWDSINITRYNPIWV | 1.1852 | No | No | No | Yes | Yes |
|  |  | 127 | DSINITRYNPIWVKT | 0.9188 | No | No | No | Yes | Yes |
|  |  | 128 | SINITRYNPIWVKTP | 0.7839 | No | No | No | Yes | Yes |

Table S5 DBoV Construct Sequence

| Properties | DBoV Construct Sequence |
| --- | --- |
| DBoV-A1 | GIINTLQKYYCRVRGGRCAVLSCLPKEEQIGKCSTRGRKCCRRKKEAAAKAKFVAAWTLKAAAAAYYNYLGPFNPLAAYKQLAPGRSAAAYFSPKEWQTLAAYKVYNIQIKAAYFQNDLTAGLAAYKQYAYITCPYAAYMVDTRDGTLGPGPGAYNQYLNKGLNPYLKGPGPGKLYFARSNKGAKRQRGPGPGEWQTLLNIAKRFRPVGPGPGAWDSINITRYNPIWVKKYLGPFNPLDNGEPVNKKKVKRALAPSLNEKQLAPKKYFARSNKGAKRQRLNPKKANAGTGGVGMSTGQWIKKDSINITRYNPIWVKTPKKEIEWEYETHFNKNWRP |
| DBoV-A2 | MAENPNIDDLPAPLLAALGAADLALATVNDLIANLRERAEETRAETRTRVEERRARLTKFQEDLPEQFIELRDKFTTEELRKAAEGYLEAATNRYNELVERGEAALQRLRSQTAFEDASARAEGYVDQAVELTQEALGTVASQTRAVGERAAKLVGIELEAAAKAKFVAAWTLKAAAAAYYNYLGPFNPLAAYKQLAPGRSAAAYFSPKEWQTLAAYKVYNIQIKAAYFQNDLTAGLAAYKQYAYITCPYAAYMVDTRDGTLGPGPGAYNQYLNKGLNPYLKGPGPGKLYFARSNKGAKRQRGPGPGEWQTLLNIAKRFRPVGPGPGAWDSINITRYNPIWVKKYLGPFNPLDNGEPVNKKKVKRALAPSLNEKQLAPKKYFARSNKGAKRQRLNPKKANAGTGGVGMSTGQWIKKDSINITRYNPIWVKTPKKEIEWEYETHFNKNWRP |
| DBoV-A3 | MAKLSTDELLDAFKEMTLLELSDFVKKFEETFEVTAAAPVAVAAAGAAPAGAAVEAAEEQSEFDVILEAAGDKKIGVIKVVREIVSGLGLKEAKDLVDGAPKPLLEKVAKEAADEAKAKLEAAGATVTVKEAAAKAKFVAAWTLKAAAAAYYNYLGPFNPLAAYKQLAPGRSAAAYFSPKEWQTLAAYKVYNIQIKAAYFQNDLTAGLAAYKQYAYITCPYAAYMVDTRDGTLGPGPGAYNQYLNKGLNPYLKGPGPGKLYFARSNKGAKRQRGPGPGEWQTLLNIAKRFRPVGPGPGAWDSINITRYNPIWVKKYLGPFNPLDNGEPVNKKKVKRALAPSLNEKQLAPKKYFARSNKGAKRQRLNPKKANAGTGGVGMSTGQWIKKDSINITRYNPIWVKTPKKEIEWEYETHFNKNWRP |
| DBoV-A4 | MAQVINTNSLSLLTQNNLNKSQSSLSSAIERLSSGLRINSAKDDAAGQAIANRFTSNIKGLTQASRNANDGISIAQTTEGALNEINNNLQRVRELSVQATNGTNSDSDLKSIQDEIQQRLEEIDRVSNQTQFNGVKVLSQDNQMKIQVGANDGETITIDLQKIDVKSLGLDGFNVNGPKEATVGDLKSSFKNVTGYDTYAAGADKYRVDINSGAVVTDAAAPDKVYVNAANEAAAKAKFVAAWTLKAAAAAYYNYLGPFNPLAAYKQLAPGRSAAAYFSPKEWQTLAAYKVYNIQIKAAYFQNDLTAGLAAYKQYAYITCPYAAYMVDTRDGTLGPGPGAYNQYLNKGLNPYLKGPGPGKLYFARSNKGAKRQRGPGPGEWQTLLNIAKRFRPVGPGPGAWDSINITRYNPIWVKKYLGPFNPLDNGEPVNKKKVKRALAPSLNEKQLAPKKYFARSNKGAKRQRLNPKKANAGTGGVGMSTGQWIKKDSINITRYNPIWVKTPKKEIEWEYETHFNKNWRP |

Table S6 Analysis of vaccine secondary structure results

| Properties | DBoV-A1 | DBoV-A2 | DBoV-A3 | DBoV-A4 |
| --- | --- | --- | --- | --- |
| alpha helix | 25.60% | 51.30% | 25.62% | 38.31% |
| beta turn | 0% | 0.67% | 5.23% | 6.13% |
| random coil | 63.10% | 48% | 69.12% | 55.56% |

Table S7 Individual percentages of CD4+ and CD8+ T cells in splenocytes from each experimental group.

| Group | CD3+CD4+ T cells (%) | CD3+CD8+ T cells (%) | CD4+/CD8+ |
| --- | --- | --- | --- |
| PBS+Adj | 45.97% | 21.33% | 2.16% |
| DBoV-A2 | 54.57% | 19.03% | 2.87% |
| DBoV-A4 | 52.33% | 17.47% | 3.0% |


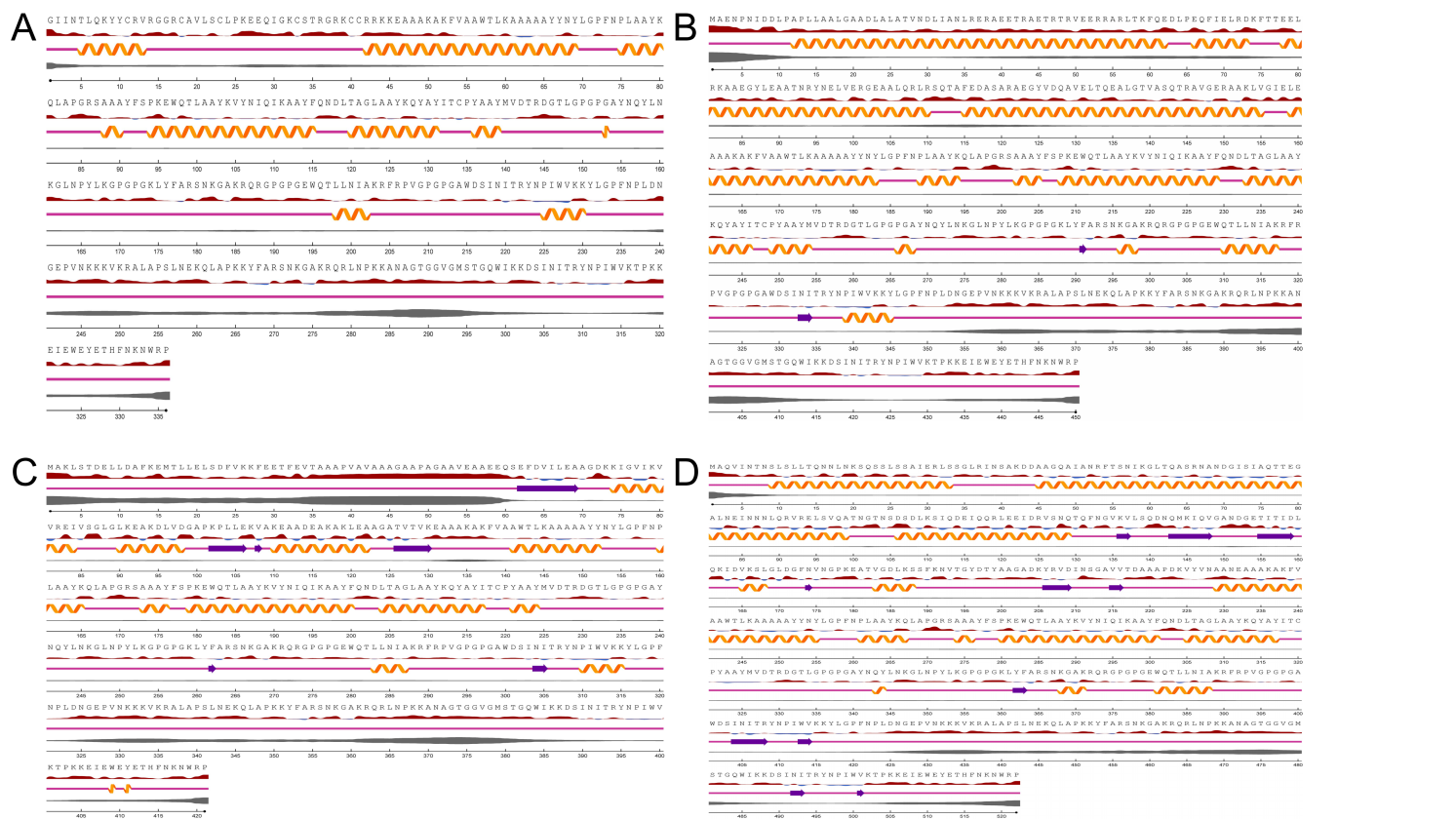


**Supplementary Figure 1.** The secondary structural features of the vaccines predicted using NetSurfP-3.0 software were as follows: DBoV-A1 contained 25.6% alpha helices, 0% beta turns, and 63.1% random coils; DBoV-A2 contained 51.3% alpha helices, 0.67% beta turns, and 48% random coils; DBoV-A3 contained 25.62% alpha helices, 5.23% beta turns, and 69.12% random coils; and DBoV-A4 contained 38.31% alpha helices, 6.13% beta turns, and 55.56% random coils

**
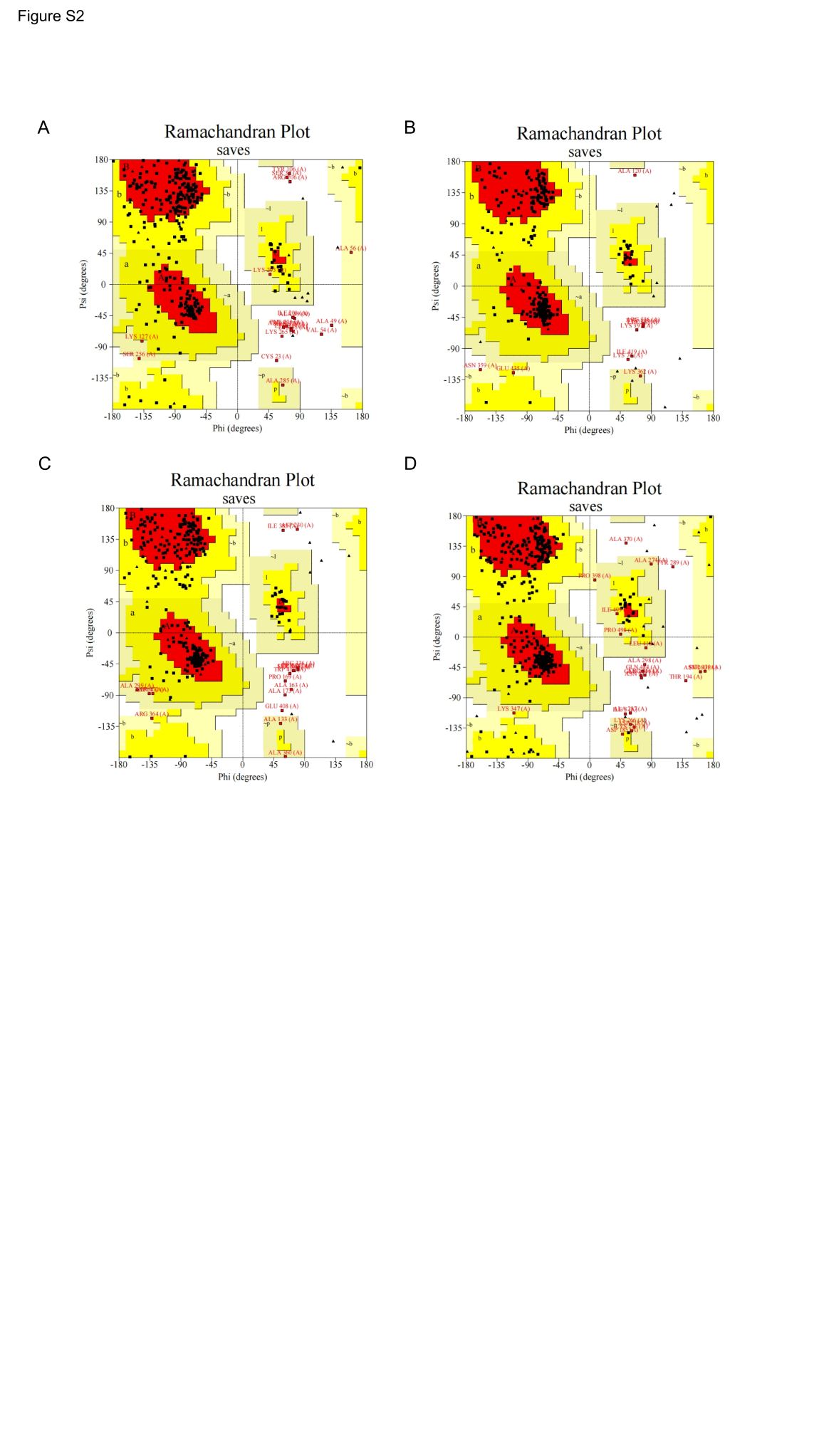
**

**Supplementary Figure 2.** (A) for DBoV-A1, 68.1%, 25.1%, and 1.8% of residueswere located in the most favoured, additionally allowed, and disallowed regions, respectively; (B) for DBoV-A2, 87.6%, 9.8%, and 0.8% of residues were located in the most favoured, additionally allowed, and disallowed regions, respectively; (C) for DBoV-A3, 79.8%, 15.7%, and 1.7% of residues were located in the most favoured, additionally allowed, and disallowed regions, respectively; (D) for DBoV-A4, 80.3%, 15.3%, and 2.2% of residues were located in the most favoured, additionally allowed, and disallowed regions, respectively.


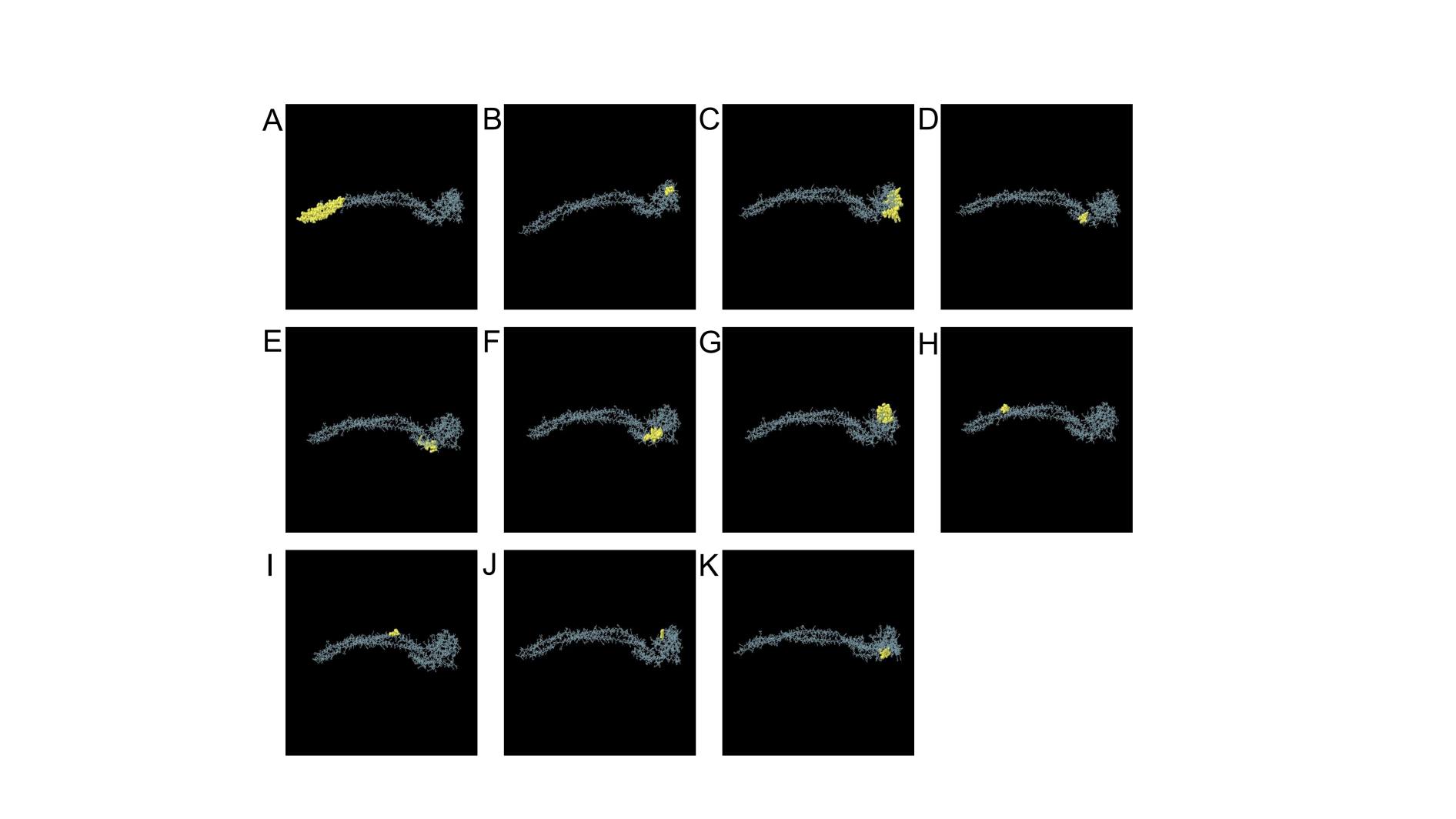


**Supplementary Figure 3.** The B-cell conformational epitopes predicted by Ellipro software were specifically targeted against DBoV-A2. DBoV-A2 comprises 213 residues, with a score ranging from 0.514 to 0.806, and the epitope span of DBoV-A2 spans 3 to 75 amino acid residues.

**
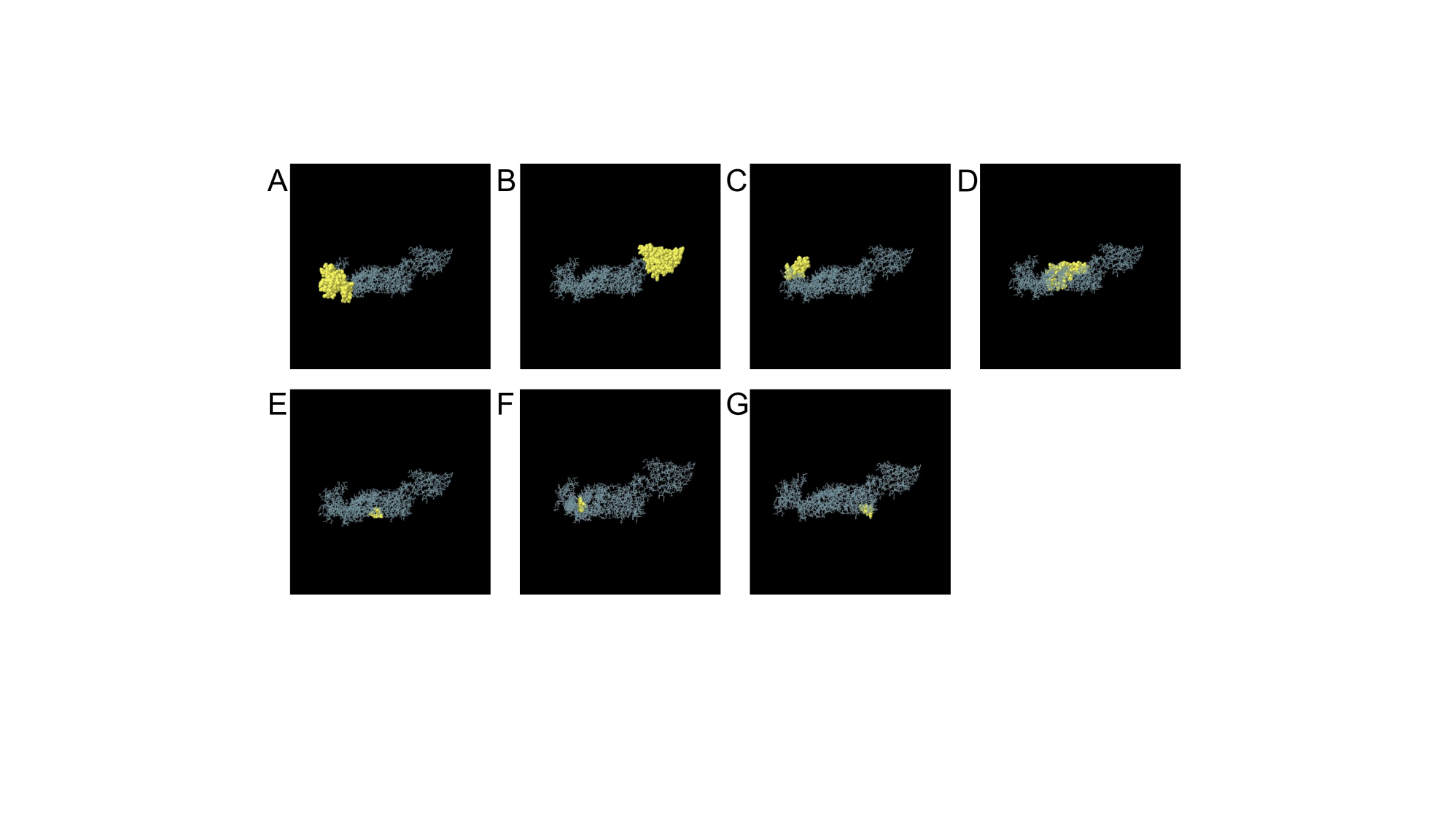
**

**Supplementary Figure 4.** The B-cell conformational epitopes predicted by Ellipro software were specifically targeted against DBoV-A4. DBoV-A4 comprises 258 residues, with a scoring range of 0.529 to 0.827. The epitope range of DBoV-A4 spans from 3 to 96 residues.
